# Mapping the landscape: A bibliometric analysis of AI and teacher collaboration in educational research

**DOI:** 10.12688/f1000research.160297.1

**Published:** 2025-02-13

**Authors:** Arvind Nain, N.S Bohra, Archana Singh, Rekha Verma, Rakesh Kumar, Rajesh Kumar

**Affiliations:** 1 Department of management, Graphic Era Deemed to be University, Dehradun, Uttarakhand, India; 2 DBS Global University, Dehradun, Uttarakhand, India; 3 Uttaranchal Institute of Management, Uttaranchal University, Dehradun, Uttarakhand, India

**Keywords:** Artificial Intelligence, Teachers, Teaching, Education, Bibliometric

## Abstract

**Background:**

This study intends to investigate the relationship between artificial intelligence and teachers’ collaboration in educational research in response to the growing use of technologies and the current status of the field.

**Methods:**

A total of 62 publications were looked at through a systematic review that included data mining, analytics, and bibliometric methods.

**Result:**

The study shows a steady increase in the field of artificial intelligence and teacher collaboration in educational research, especially in the last few years with the involvement of the USA, China, and India. Education and information technology are the main contributors to this field of study, followed by an international review of open and distance learning research. The Scopus database is used in this study to find publication trends, important papers, major themes, and keywords. It also looks at target concepts and futuristic themes.

**Conclusions:**

Over a three-year period, the average citation value is 12.44%. The education system, learning, e-learning, sustainability, COVID-19 issues, team challenges, organizational conflicts, and digital transformation are just a few of the topics it significantly contributes to. The study acknowledges its limitations and considers potential avenues for additional research. The results also emphasize important gaps in the literature, highlighting the necessity for more research. This information can help develop strategic approaches to address issues and take advantage of opportunities relating to artificial intelligence and teacher collaboration in higher education and research. The study’s ultimate goal is to offer guidance for tactics that promote teachers’ cooperation in educational research and the development of artificial intelligence.

## 1. Introduction

Artificial intelligence is a fast evolving technology, which has the potential to transform all aspects of our social relationships. Artificial intelligence (AI) has become more prevalent. Technology is infiltrating so many aspects of our life and we have no option but to face its consequences ( Rai & Mishra, 2022). AI is viewed as a constantly evolving subject that spans a wide variety of topics apart from computational thinking. AI encompasses essentially just the comprehension of natural language and machine learning, but also broader skills such as logical reasoning, sense-making, appraisal, and troubleshooting ( Smith & Humphreys, 2006; Chen et al., 2020). As AI increases many aspects of education, it also raises important considerations about the ever-changing role of the educator’s job ( Pelletier et al., 2021). Teachers must adapt and embrace technology improvements while maintaining the fundamental principles of good teaching and personal connection. Concerns about job displacement, the necessity for technical skill and the ethical issues concerning AI in education highlight the complex interaction between educators and evolving technology ( Zawacki Richter et al., 2019). Artificial intelligence involves the development of computer systems that mimic human intelligence. This entails tasks such as learning, reasoning, problem solving, and adapting to new information. AI applications range from speech recognition and image analysis to self-driving cars and advanced decision-making systems. AI tools evaluate the personal data of each student, modifying the curriculum and pace to meet different learning requirements. This open environment allows students to learn at their own pace and in the manner that best fits them. AI also makes it easier to provide real-time feedback, which helps teachers and students alike pinpoint areas that need work. AI also makes it possible to create interactive simulations, virtual instructors, and interesting information, all of which improve learning in general. Artificial Intelligence (AI) in education enhances learning results, making it more accessible, effective, and customized to each student’s requirements by creating equilibrium between technology and human interaction ( Wang et al., 2022). Artificial Intelligence in Education (AIEd) is the use of AI technology, such as intelligent tutoring systems, chatbots, robots, and automated evaluation tools, to improve and assist education. AIEd’s great promise rests in its capacity to customize and modify education for students, improve instructors’ understanding of learning processes, and provide machine-supported questions and fast observations, creating education engaging and accessible at any time and from any location. The significance of studying and applying AI in education is demonstrated by a variety of national and international initiatives and research projects ( Metli, 2023). For illustration, during the year 2019, the Chinese government developed a strategic education modernization program to support deeper incorporation of artificial intelligence within schooling as well as in teacher’s professional growth events connected to AI and AIEd ( Chiu, 2021; Chiu et al., 2022; Xia et al., 2022). While Artificial Intelligence (AI) develops to be integrated into classrooms, instructors’ work happiness becomes a priority. AI solutions, such automated grading systems and tailored learning platforms, have the potential to improve efficiency and creativity. However, the changing role of instructors and the incorporation of technology present obstacles. Considering the advantages of AI while maintaining human interaction in education is critical for growing work satisfaction among school instructors in this rapidly changing technology world. In the United States, materials and funding have been made available to selected organizations and institutions for exploring and creating personalized education platforms powered by artificial intelligence, These efforts have enormous potential to improve student achievement by increasing intellectual involvement and addressing educational inequities, especially for deprived students ( Boninger et al., 2020). The higher intellectual engagement also has addressed the educational problems among some students with different level ( Williamson & Eynon, 2020).

## 2. Literature review

### 2.1 Artificial intelligence

Artificial intelligence is revolutionizing education by automating monotonous tasks, customizing learning experiences, and providing analytical data on student performance ( Van der maaten & Hinton, 2008). Educational institutions are increasingly using AI-powered solutions to save costs, improve learning outcomes, and provide each student with a personalized learning experience. This technology also simplifies administrative procedures, allowing teachers to concentrate on teaching. Furthermore, AI brings novel kinds of interaction, which include interactive simulations and learning platforms as well rendering education more available and successful for all kinds of students. Across learning, artificial intelligence is growing to provide innovative instructional and educational solutions that are now being tested in a variety of scenarios. Computer scientists have made significant contributions to the research and development of artificial intelligence for educational applications ( Chen et al., 2020; Zawacki-Richteret al., 2019; Williamson & Eynon, 2020).

### 2.2 Artificial intelligence and Teachers Collaborations

Teachers play a critical role in an artificial intelligence-based system of learning that extends beyond traditional instruction. While AI provides tailored education and administrative efficiency, instructors provide the crucial human components of empathy, comprehension, and moral direction. They analyze and enhance AI-generated insights with expert judgment, personalizing training to each student’s unique requirements. Teachers also serve as facilitators, teaching students through AI tools and developing critical thinking abilities to identify and examine the information offered by AI. Furthermore, they play an important role in developing social and emotional abilities that AI cannot imitate. Therefore, in the present day, instructors transition from solo information producers to guides and mentors in an improved collaborative and participatory educational experience. The practical uses of machine learning in education varied and crucial ( Luo et al., 2022). For example, personalized learning systems like as Dream Box Learning employ AI to tailor lessons in mathematics to each student’s learning style and speed, resulting in more effective and tailored training. In language learning, platforms such as Duolingo use AI to create individualized activities that improve language ability more effectively. Powered by AI chatbots, which include Georgia State University’s “Pounce,” assist students with admission and financial aid issues while reducing administrative operations. AI-powered programs, such as Microsoft’s Seeing AI app, convert visual information into audio feedback for visually challenged pupils, increasing access to instructional content. These apps highlight how artificial intelligence may improve education by making it more customized, efficient, and accessible.

Many modern commercial AI systems designed for schooling. like artificial intelligent tutoring systems (ITS), have a focus on offering automated, adaptive, and individualized teaching, an approach that we shall go over in further depth below. Because of the deep historical connection between AI and behavioral science ( Gardner, 1985), numerous important artificial intelligence in education have been developed on intellectual architectures, which assume that the human brain operates as a processor of knowledge. According to this approach, learning is primarily concerned with the development of problem-solving skills, which are dependent on the presence of necessary information structures within the human mind ( Carvalho et al., 2022a, 2022b). The efficiency of solving the problem depends on experience and knowledge have been discussed by Alves et al. (2022) and Kusumastuti et al. (2023) in their research as well. Given AI’s potential for improving education, educational scholars, policymakers, and practitioners have turned their attention to AIEd. However, the early focus of study was mostly on engineering factors, including the invention of novel algorithms and the improvement of artificial intelligence and deep learning approaches. In contrast to other areas of educational technology, such as videogames and blended learning, study in AI in Education is disorganized and lacks a unified framework. The influence of AI over education is yet not certain ( Holmes & Tuomi, 2022), Further research is required to determine the best scenarios and strategies for using these developing technologies into education. Limited awareness of technology innovations creates barriers to their effective integration in educational institutions ( Hussain, 2018). As an outcome, additional analyses will be needed to incorporate the material, offer a thorough picture of the opportunities and problems associated with Artificial Intelligence in Education (AIE), and identify areas for future study. As a result, there has been a significant increase in the overall number of journal papers on this subject. To illustrate, Zhai (2021) had done in research synthesis concentrating on developments in artificial intelligence educational technology; hence addressed to mostly engineering elements ( King et al., 2022; Bozkurt et al., 2021). Examined prevailing course in AI educational articles, such as subject areas, regional dissemination and textual data sample. Other reviews focused on specialized subjects, such as language learning, mathematical concepts and medical studies ( Karaca et al., 2021), regarding some educational tasks, such as evaluation ( Jiménez-Hernández et al., 2020) also in specific technology or programs, like assisting computerized robot, machine learning ( Muñoz et al., 2022; Wang et al., 2023; Papadopoulos et al., 2022). However, they are review research studies concentrated over a single major area (learning, teaching, evaluation, or management) or a specific educational result. We would need more comprehensive approach which will analyze the function of artificial intelligence in education ( Nigam et al., 2021; Chiu et al., 2023).

The COVID-19 epidemic has forced a rapid transition from traditional education to online learning, with artificial intelligence (AI) emerging as a key role ( Wang, 2022; Mhlanga, 2022; Stadlman et al., 2022). Online platforms and AI technologies have the ability to provide a more individualized educational experience, which increases efficacy. Smart assistants powered by AI automate administrative duties for instructors, such as attendance tracking and lesson planning. This efficiency gives instructors additional time and energy, allowing teachers to concentrate on individual student attention. AI integration in education reacts to the changing demands of teaching within a digital age, increasing both instructor effectiveness and learning for students experiences ( Nemorin et al., 2023). ChatGPT, designed by OPENAI2, has provoked an educational watershed moment, raising ethical, technological, and practical challenges that impact the learning and teaching interaction ( Adiguzel et al., 2023).

## 3. Methods

**Figure 1.**
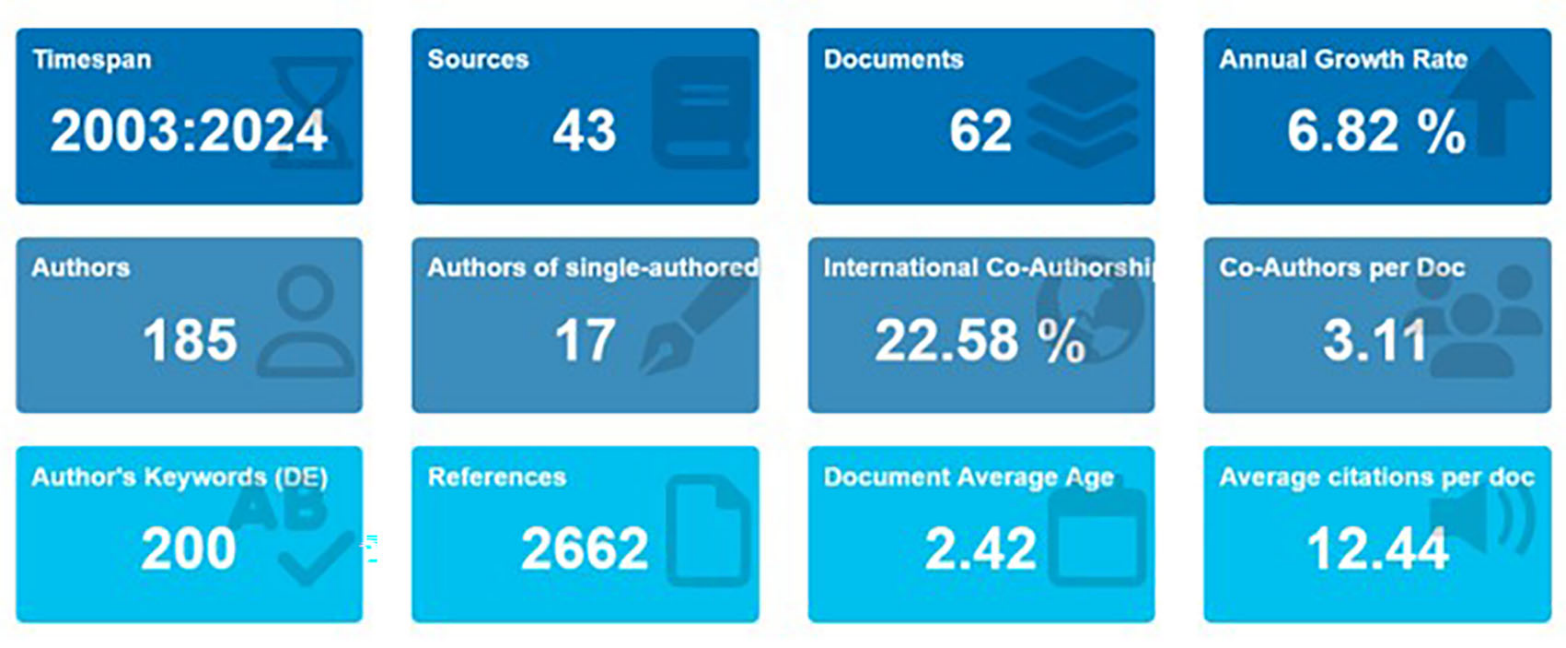
Main information. Source: BiblioShiny.

A bibliometric analysis aims to address specific research questions using a clear, systematic, and replicable search strategy ( Donthu et al., 2021). This process involves identifying relevant studies, synthesizing data, and analyzing trends, such as the annual publication rates of articles ( Lin et al., 2013). The study focuses on exploring the literature on the use of artificial intelligence (AI) in education over the past 40 years, starting from 1984. The primary objectives are to answer the following questions: Which entities—such as research institutions, universities, countries, regions, and research communities—are the leading contributors to AI research in education? What is the intellectual, co Figure 1 displays the synthesised bibliometric data, which offers a comprehensive summary of the field’s current gaps or inconsistencies as well as research trends and real-world applications. This study provides insights into the issue and its evolution by methodically mapping 644 publications on AI in education.nceptual, and social framework of this research? How has the field of AI in education evolved over time?

## 4. Results

### A. Data synthesis

Starting in 2003, this study looks at the literature on teacher cooperation and artificial intelligence (AI) in education during the previous 20 years. The following important questions are the focus of the study: Which organisations are the main contributors to research on artificial intelligence and teacher cooperation in education, including research institutes, universities, nations, regions, and research communities? Which conceptual, intellectual, and social frameworks are influencing this study? What changes have you seen over the years in the research of AI and teacher collaboration in education? A detailed summary of research trends and advancements in AI and teacher collaboration in education is shown in Figure 1, which presents the results of bibliometric analysis.

### B. The patterns of article publication over time

**Figure 2.**
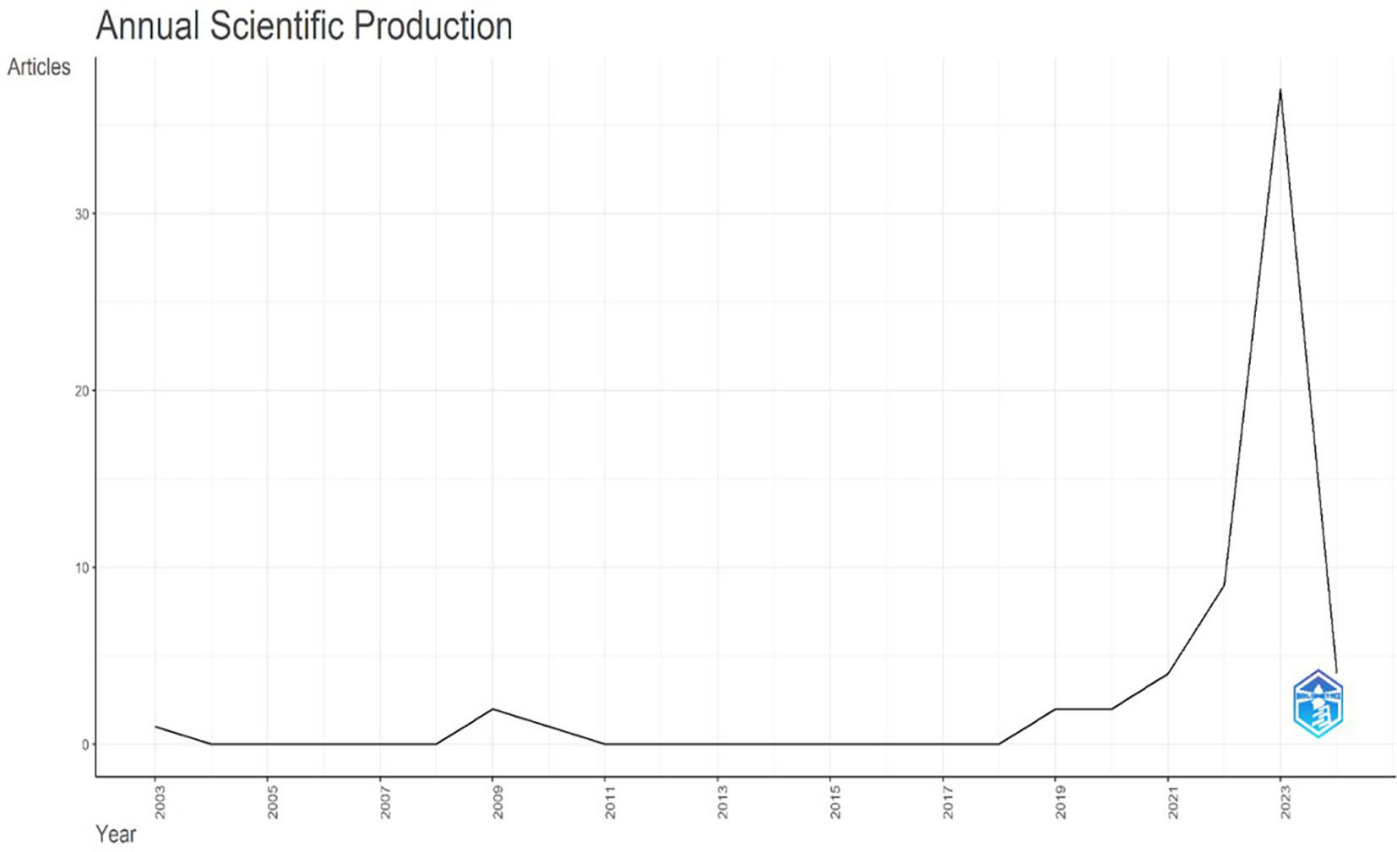
Annual scientific production. Source: BiblioShiny.

The distribution of papers on AI and teacher collaboration in education for the last 20 years (2003–2024) is shown in Figure 2. According to the data, research activity peaked in 2021 with the publication of 15 documents, and then significantly increased to 37 publications in 2023. It is important to remember that the 2024 numbers only cover the first five months of the year; by the end of the year, the total is anticipated to have increased. Prior to 2022, the field’s publication output fluctuated, showing inconsistent research activity, despite growing interest in AI and teacher collaboration in education. Publications about AI in education are growing at a pace of 6.82 per year. The average annual citations are displayed in Figure 2 and exhibit an increasing tendency over time, but with considerable irregularity

### C. Source growth

**Figure 3.**
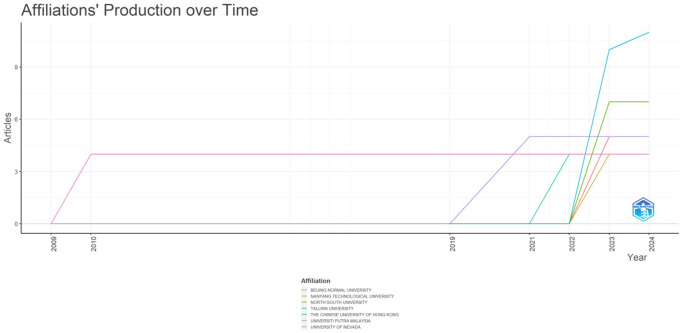
Affiliations production over time. Source: BiblioShiny.

The number of publications linked to different universities annually in Figure 3 shows journal contributions to research on AI and teacher cooperation in education. In the line chart, each university is represented by a different colour, and the study focusses on the top five because of their noteworthy contributions.

With publications in this area starting in 2022 and rising yearly after 2023, the Chinese University of Hong Kong and North South University have shown steady and significant development in their outputs. Since 2003, however, the other universities have contributed very little to this field of study.

### D. Word Cloud, and Treemap

**Figure 4.**
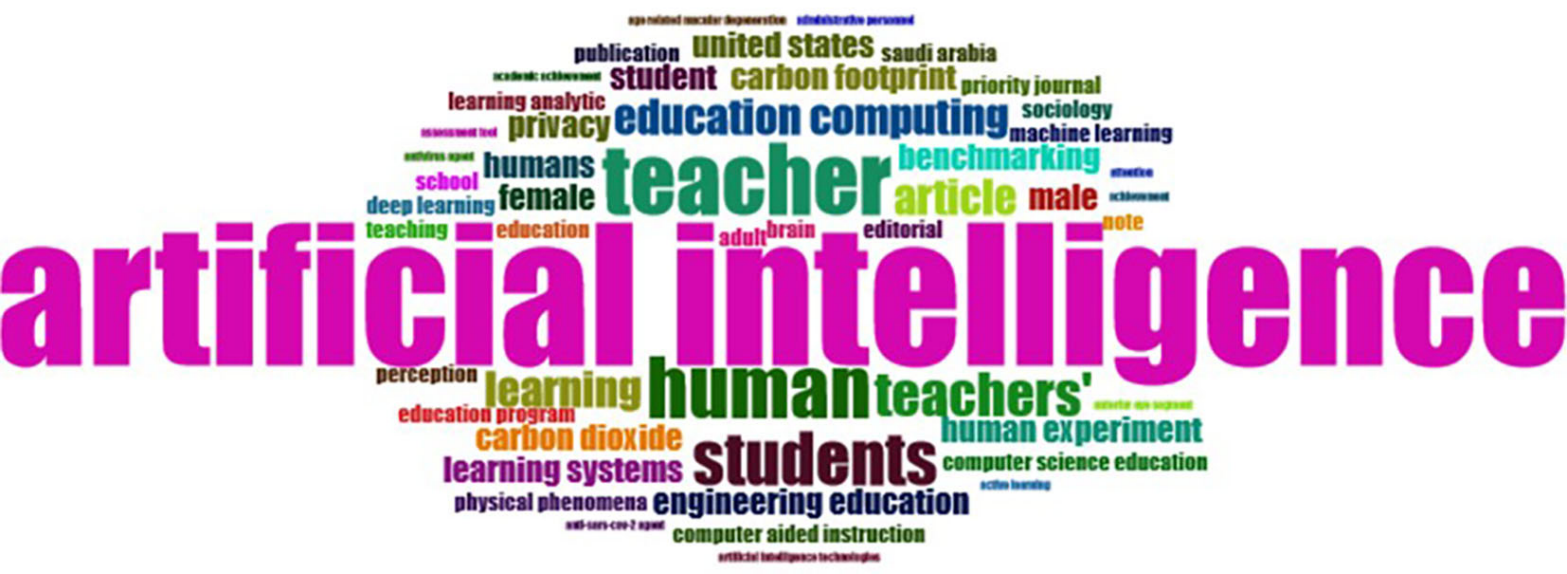
Word Cloud. Source: BiblioShiny.

The number of articles that journals associate with different universities annually illustrates their contributions to research on AI and teacher cooperation in education ( Figure 4). Each university is represented by a different colour code in the line chart, and the top five universities are highlighted in the analysis because of their noteworthy contributions. Beginning in 2022 and growing yearly after 2023, the Chinese University of Hong Kong and North South University have demonstrated consistent and noteworthy growth in this area. Comparatively, since 2003, other universities have contributed very little to this field of study.

### E. Word growth

**Figure 5.**
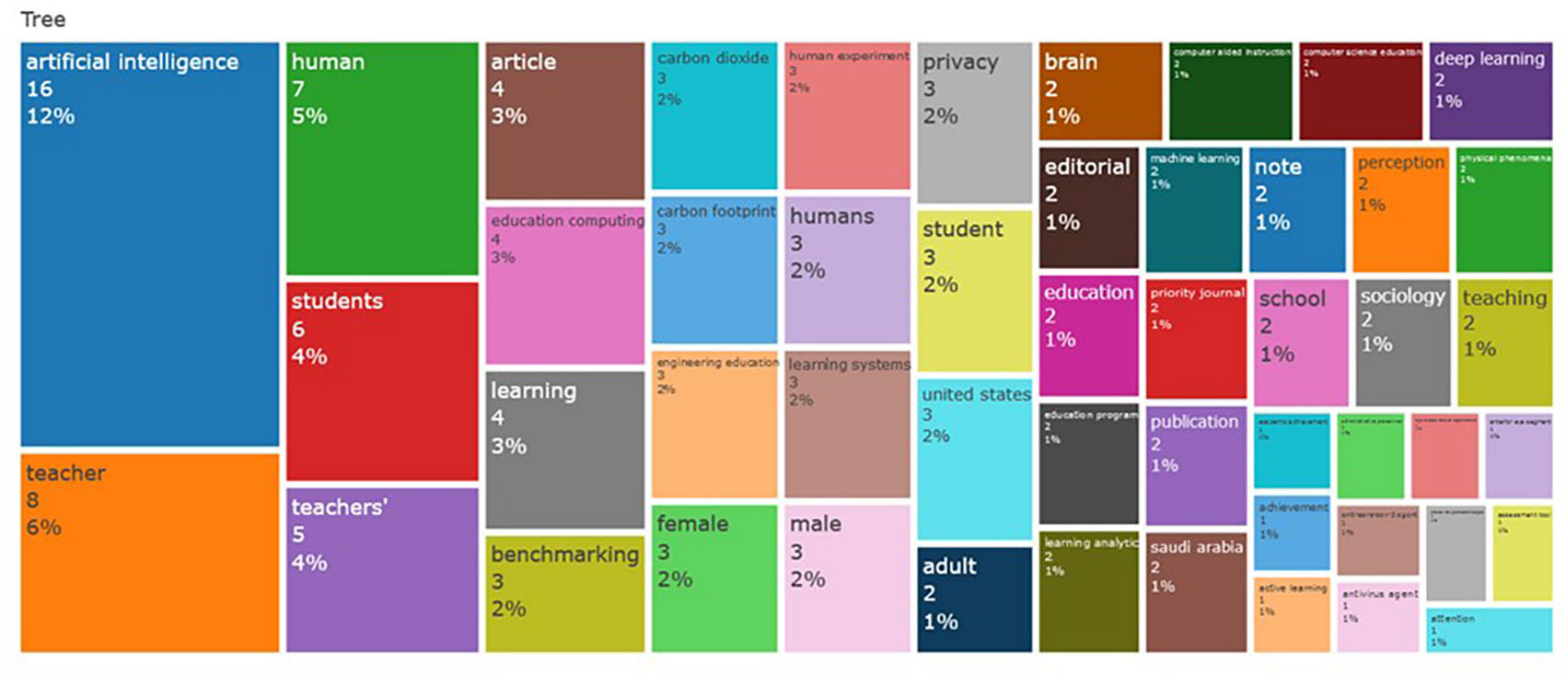
Treemap. Source: BiblioShiny.

Examining word growth provides important information on how literary terminology has changed throughout time. As shown in Figure 5 AI and teacher cooperation in education, where understanding the development of the field is aided by monitoring the introduction and impact of key phrases. learning system, employee, students, and educational computing are some of the first terms in this domain. For professionals, analysts, and researchers, this analysis is an essential tool that helps them spot patterns and gain insightful knowledge. Analysing word frequency over time reveals trends that can help influence strategic planning and decision-making in a variety of situations.

### F. Country-specific production and collaborations

**Figure 6.**
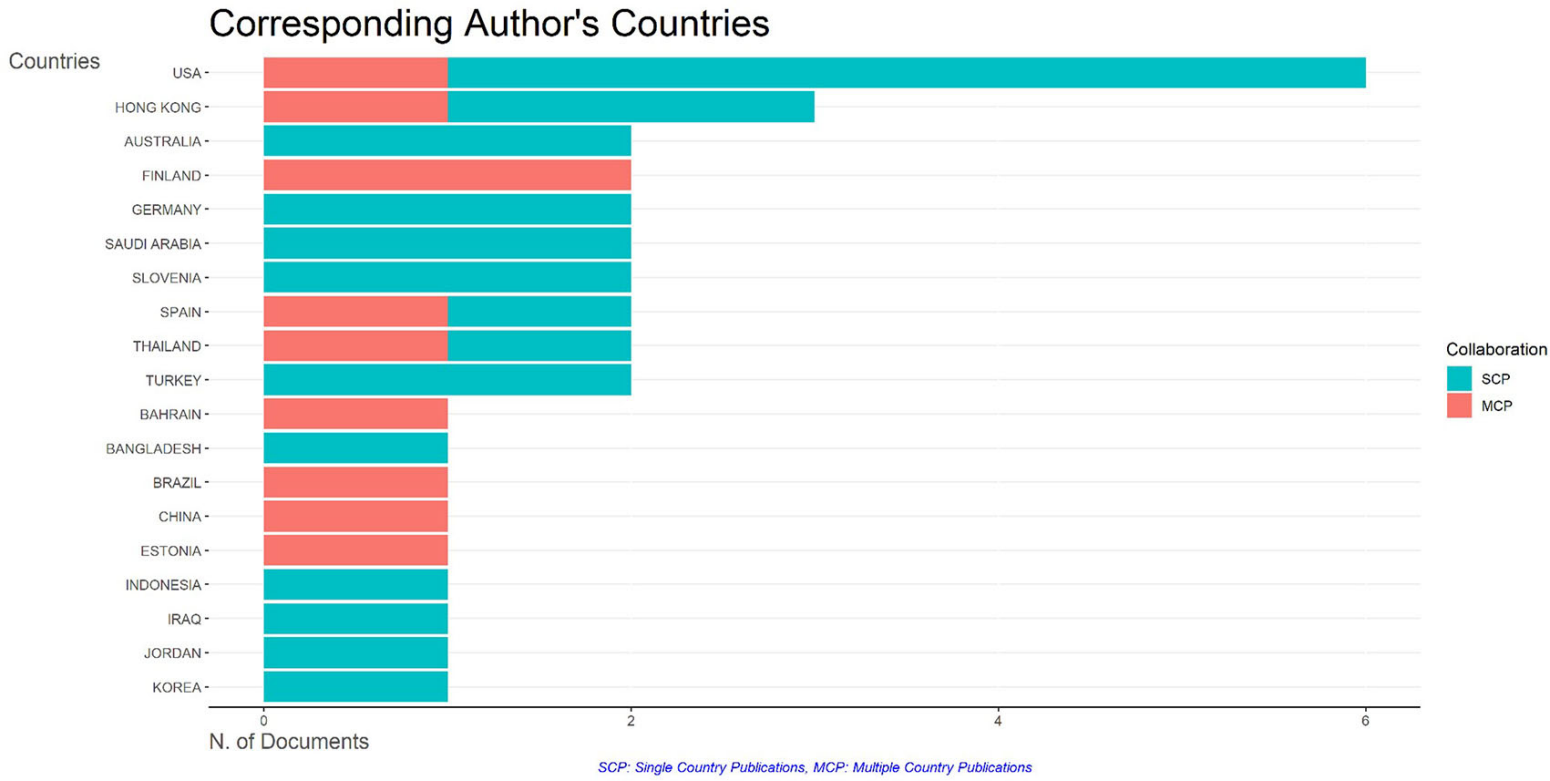
Most productive country. Source: BiblioShiny.

The cooperation globe map displays a graphical depiction of author affiliations by nation. The two major nations with the closest international links are the USA and Hong Kong. The number of publications these countries produce is directly correlated with the intensity of these ties. The global collaboration map for AI and teacher collaboration research in education is shown in Figure 6.

The USA, a leading contributor to this subject, works closely with countries including Australia, Hong Kong, and the United Kingdom. Researchers from the USA show a great propensity to cultivate worldwide relationships, hence increasing cooperative efforts in AI and education research, despite linguistic variations among participating countries.

The USA, a leading contributor to this subject, works closely with countries including Australia, Hong Kong, and the United Kingdom. Researchers from the USA show a great propensity to cultivate worldwide relationships, hence increasing cooperative efforts in AI and education research, despite linguistic variations among participating countries.

### G. Trend topics

**Figure 7.**
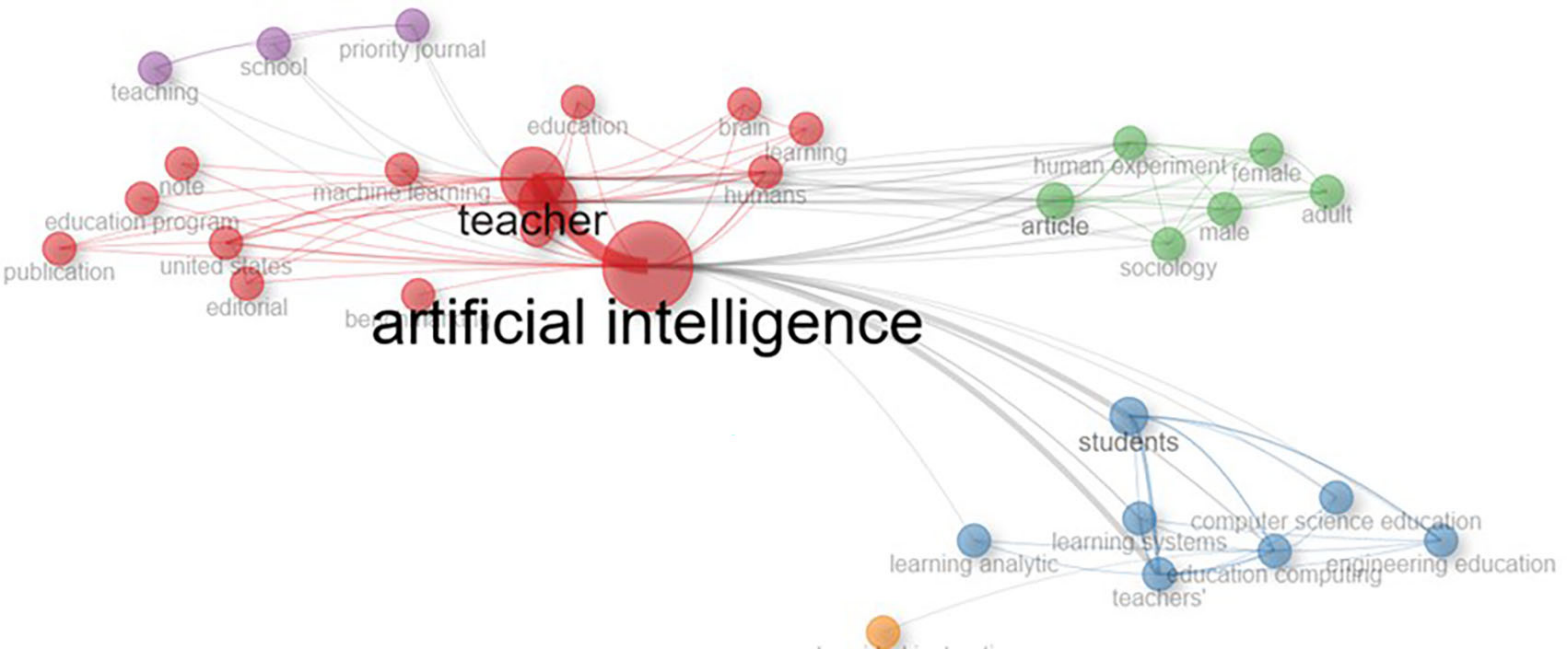
Co-occurrence network. Source: Vosviwer.

A quantitative analysis of the influence of scholarly literature is shown in Figure 7, possibly using bibliometric techniques. This assessment takes into account measures like publications and citations, offering a quantifiable indication of the impact and prominence of research results. Important terms like “Artificial Intelligence,” “Teacher,” “Teachers,” “Students,” and “human,” which probably reflect major issues in the literature, are included in the study. According to the results, “Artificial Intelligence” became the most popular topic, especially in 2023, indicating a major emphasis on AI and its cooperation with educators.

This demonstrates how research trends are dynamic and underscores the necessity of continuous observation. It is advised that researchers remain alert to new developments and modify their focus to meet changing goals and problems.

Similarly, Table 1 assesses the influence of scholarly work using a quantitative method that may include bibliometric tools. Similar to Figure 7, it looks at publication data and citations to gauge the scope and importance of research. Additionally, it uses words like “Artificial Intelligence,” “Teacher,” “Teachers,” “Students,” and “human,” which are in line with important literary themes. The findings demonstrate the increasing convergence of artificial intelligence and education, confirming that “Artificial Intelligence” was the most popular topic in 2023. This emphasises how crucial it is to remain aware of changing research environments and match studies with new fields of interest.

**Table 1.** Word frequency per year.

item	freq	year_q1	year_med	year_q3
teacher	8	2018	2022	2023
students	6	2022	2022	2023
artificial intelligence	16	2022	2023	2023
human	7	2022	2023	2023
teachers’	5	2023	2023	2023

Source: BiblioShiny.

### H. The co-occurrence network analysis

Different theme streams in the literature on AI-teacher collaboration in education are revealed by the co-occurrence network analysis. Colour-coded clusters are used to graphically represent these streams. Because of its high centrality, the red cluster is considered the central cluster, indicating that certain keywords within it are more important to the network as a whole. Other subjects in the literature are probably influenced and connected by these important terms, which serve as connectors.

Thematic relationships are represented by the interconnecting purple and green clusters in Figure 7, which show that keywords commonly occur together in these clusters. This suggests that the concepts these keywords reflect have a common theme or conceptual overlap. On the other hand, because they are not connected by the main linking streams, the yellow-orange and grey clusters appear to reflect more discrete subjects. These clusters’ keywords have a weaker connection to the main ideas.

Scholars and researchers can identify important subjects and areas of interest by using information from the co-occurrence network. The choice of study themes, the development of research questions, and an emphasis on subjects that are crucial or closely related in the literature are all supported by this analysis. Furthermore, as similar topics inside the network give academics from many domains a starting point for collaboration, the relationships between clusters may also point to prospects for interdisciplinary cooperation.

## 5. Discussion

This study indicates that while this industry’s publication count has grown consistently since 2019, a bigger growth occurred in 2024, which was followed by an exponential rise after 2019. In terms of AI and teacher collaboration in Education, the United States far outpaces China. The recent publication of numerous highly referenced articles suggests an increase in the quantity of academic publications on the function of AI and teacher collaboration in Education as well as a change in the focus of some of this research. As a consequence, the industry seems to be developing significantly and moving forward. Researchers are now working to have a better understanding of how AI and teacher collaboration could influence Education more sustainably. The results of this study show that authorship and institutional location affect the level of interest in AI and teacher collaboration in Education on a worldwide scale. Unexpectedly, the US has the most authors and colleges, with Hongkong coming in second. The data clearly shows the geographic variety of the AI and teacher collaboration in Education in terms of institutional affiliations and authorships. Geographic variety is important because different areas may have different views on the role that AI should play with teachers in education. The fact that the research suggests that the focus is still on how AI functions in teacher collaboration in respond to various educations programmes inside educational institute or whether particular AI system have an influence on narrowly defined education system is a potentially worrisome aspect. Less focus has been paid to distance educations and the importance of sustainability in AI and teacher collaboration in Education systems, which emphasises how challenging it is to incorporate sustainability into Education.

## 6. Conclusion

Through a systematic review, this study delved into the realm of artificial intelligence and teacher collaboration in Education. The research revealed a growing interest in the field and a diverse range of applications of artificial intelligence technologies, highlighting the need for a comprehensive examination of their utilization from various perspectives. The findings underscored the significant reliance on artificial intelligence technologies, pointing towards a future shaped by algorithmic scenarios. Drawing upon the insights garnered from the reviewed publications, the study identified several implications for future research endeavors. Firstly, it was observed that a majority of the artificial intelligence applications in Education predominantly focus on technical aspects, disregarding crucial factors such as pedagogy, curriculum, and instructional/learning design. Secondly, despite the utilization of human-generated data in artificial intelligence technologies, there is a notable absence of regulations regarding the ethical usage of this data. To address this gap, future research could concentrate on exploring this issue and advocating for the development of policies and strategies. Educational institutions must prioritize the establishment of a human-centered approach to online learning that effectively harnesses the benefits of artificial intelligence technologies.

### Ethics and consent

Ethical approval and consent were not required.

## Data Availability

Zenodo: Mapping the Landscape: A Bibliometric Analysis of AI and Teacher Collaboration in Educational Research. Doi: 10.5281/zenodo.14706394 ( https://doi.org/10.5281/zenodo.14706394) ( Nain, 2025). This project contains the following extended data:
• Mapping the Landscape A Bibliometric Analysis of AI and Teacher Collaboration in Educational Research.csv Data is available under the terms of the Creative Commons Zero v1.0 Universal

## References

[ref16] Adiguzel T Kaya MH Cansu FK : Revolutionizing education with AI: Exploring the transformative potential of ChatGPT. *Contemp. Educ. Technol.* 2023;15(3). 10.30935/cedtech/13152

[ref17] Alves JL Nadae J de Carvalho MM de : Knowledge management enablers and barriers: exploring the moderating effect of communication barriers. *Int. J. Manag. Proj. Bus.* 2022;15(7):1091–1122. 10.1108/IJMPB-02-2022-0047

[ref32] Boninger F Molnar A Salda˜na C : *Big claims, little evidence, lots of money: The reality behind the Summit Learning Program and the push to adopt digital personalized learning platforms.* Boulder, CO: National Education Policy Center;2020. nepc.colorado.edu/publication/summit-2020.

[ref18] Bozkurt A Karadeniz A Baneres D : Artificial intelligence and reflections from educational landscape: A review of AI studies in half a century. *Sustainability (Switzerland).* 2021;13(2):1–16. 10.3390/su13020800

[ref19] Carvalho L Martinez-Maldonado R Tsai YS : How can we design for learning in an AI world? *Comput. Educ.: Artif. Intell.* 2022a;3(February):100053. 10.1016/j.caeai.2022.100053

[ref20] Carvalho RP Marchiori CFN Brandell D : Artificial intelligence driven in-silico discovery of novel organic lithium-ion battery cathodes. *Energy Storage Mater.* 2022b;44(October 2021):313–325. 10.1016/j.ensm.2021.10.029

[ref33] Chen X Xie H Zou D : Application and theory gaps during the rise of artificial intelligence in Education. *Comput. Educ.: Artif. Intell.* 2020;1:100002. 10.1016/j.caeai.2020.100002

[ref35] Chiu TKF : A holistic approach to Artificial Intelligence (AI) curriculum for K- 12 schools. *TechTrends.* 2021;65:796–807. 10.1007/s11528-021-00637-1

[ref34] Chiu TKF Meng H Chai CS : Creation and evaluation of a pre-tertiary Artificial Intelligence (AI) curriculum. *IEEE Trans. Educ.* 2022;65(1):30–39. 10.1109/TE.2021.3085878

[ref21] Chiu TKF Xia Q Zhou X : Systematic literature review on opportunities, challenges, and future research recommendations of artificial intelligence in education. *Comput. Educ.: Artif. Intell.* 2023;4(December 2022):100118. 10.1016/j.caeai.2022.100118

[ref7] Donthu N Kumar S Mukherjee D : How to conduct a bibliometric analysis: An overview and guidelines. *J. Bus. Res.* 2021;133:285–296. 10.1016/j.jbusres.2021.04.070

[ref15] Dutt A Ismail MA Herawan T : A systematic review on educational data mining. *IEEE Access.* 2017;5:15991–16005. (accessed 22 July 2019). 10.1109/ACCESS.2017.2654247 Reference Source

[ref12] Feng X Wei Y Pan X : Academic emotion classification and recognition method for large-scale online learning environment—Based on A-CNN and LSTM-ATT deep learning pipeline method. *Int. J. Environ. Res. Public Health.* 1941;17:17. 10.3390/ijerph17061941 PMC7142864 32188094

[ref44] Gardner H : Frames of mind: The theory of multiple intelligences. *Basic Books.* 1985.

[ref23] Holmes W Tuomi I : State of the art and practice in AI in education. *Eur. J. Educ.* 2022;57(4):542–570. 10.1111/ejed.12533

[ref45] Hussain S : Education 4.0 made simple: Ideas for teaching. *Int. J. Literacy Educ.* 2018;6(3):92–98. 10.7575/aiac.ijels.v.6n.3p.92

[ref24] Jiménez-Hernández D González-Calatayud V Torres-Soto A : Digital competence of future secondary school teachers: Differences according to gender, age, and branch of knowledge. *Sustainability (Switzerland).* 2020;12(22):1–16. 10.3390/su12229473

[ref26] Karaca O Çalışkan SA Demir K : Medical artificial intelligence readiness scale for medical students (MAIRS-MS) – development, validity and reliability study. *BMC Med. Educ.* 2021;21(1):112–119. 10.1186/s12909-021-02546-6 33602196 PMC7890640

[ref36] King J Holmes R Burkholder S : Advancing nature-based solutions by leveraging Engineering With Nature ^®^ strategies and landscape architectural practices in highly collaborative settings. *Integr. Environ. Assess. Manag.* 2022;18(1):108–114. 10.1002/ieam.4473 34101357

[ref27] Kusumastuti R Silalahi M Sambodo MT : Understanding rural context in the social innovation knowledge structure and its sector implementations. *Manag. Rev. Q.* 2023;73(4):1873–1901. 10.1007/s11301-022-00288-3

[ref4] Lin CF Yeh Y Hung YH : Data mining for providing a personalized learning path in creativity: An application of decision trees. *Comput. Educ.* 2013;68:199–210. 10.1016/j.compedu.2013.05.009

[ref14] Luo Y Han X Zhang C : Prediction of learning outcomes with a machine learning algorithm based on online learning behaviour data in blended courses. *Asia Pac. Educ. Rev.* 2022;25:267–285. 10.1007/s12564-022-09749-6

[ref9] Maaten L van der Hinton G : Visualizing data using t-SNE. *J. Mach. Learn. Res.* 2008;9:2579–2605.

[ref25] Metli A : Articles on Education and Artificial Intelligence: A Bibliometric Analysis Articles on Education and Artificial Intelligence: A Bibliometric Analysis. *J. Soc. Sci. Educ. (JOSSE).* 2023;6:279–312. 10.53047/josse.1352197

[ref28] Mhlanga D : The Role of Artificial Intelligence and Machine Learning Amid the COVID 19 Pandemic: What Lessons Are We Learning on 4IR and the Sustainable Development Goals. *Int. J. Environ. Res. Public Health.* 2022;19(3). 10.3390/ijerph19031879 35162901 PMC8835201

[ref29] Muñoz JLR Ojeda FM Jurado DLA : Systematic Review of Adaptive Learning Technology for Learning in Higher Education. *Eurasian J. Educ. Res.* 2022;2022(98):221–233. 10.14689/ejer.2022.98.014

[ref37] Nain A : Mapping the Landscape: A Bibliometric Analysis of AI and Teacher Collaboration in Educational Research.[Data set]. *Zenodo.* 2025. 10.5281/zenodo.14706394 PMC12082066 40386461

[ref39] Nemorin S Vlachidis A Ayerakwa HM : AI hyped? A horizon scan of discourse on artificial intelligence in education (AIED) and development. *Learn. Media Technol.* 2023;48(1):38–51. 10.1080/17439884.2022.2095568

[ref38] Nigam A Pasricha R Singh T : A systematic review on ai-based proctoring systems: Past, present and future. *Educ. Inf. Technol.* 2021;26(5):6421–6445. 10.1007/s10639-021-10597-x 34177348 PMC8220875

[ref40] Papadopoulos I Koulouglioti C Papadopoulos C : *Transcultural Artificial Intelligence and Robotics in Health and Social Care.* Academic Press;2022.

[ref5] Pelletier K Brown M Brooks DC : Educause Horizon Report Teaching and Learning Edition. *Educause.* 2021. (accessed on 18 December 2022). Reference Source

[ref3] Prinsloo P : Fleeing from Frankenstein’s monster and meeting Kafka on the way: Algorithmic decision-making in higher education. *E-Learn.* 2017;14:138–163.

[ref1] Rai A Mishra A : The Role of Artificial Intelligence in the Automation of Human Resources. *Adoption and Implementation of AI in Customer Relationship Management.* Jan 2022. 10.4018/978-1-7998-7959-6.ch011

[ref10] Smith AE Humphreys MS : Evaluation of unsupervised semantic mapping of natural language with Leximancer concept mapping. *Behav. Res. Methods.* 2006;38:262–279. 10.3758/BF03192778 16956103

[ref30] Stadlman M Salili SM Borgaonkar AD : Artificial Intelligence Based Model for Prediction of Students’ Performance: A Case Study of Synchronous Online Courses During the COVID-19 Pandemic. *J. STEM Educ.* 2022;23(2):39–46. Reference Source

[ref11] Wang C : Emotion recognition of college students’ online learning engagement based on deep learning. *Int. J. Emerg. Technol. Learn.* 2022;17:110–122. 10.3991/ijet.v17i06.30019

[ref31] Wang S Christensen C Cui W : When adaptive learning is effective learning: comparison of an adaptive learning system to teacher-led instruction. *Interact. Learn. Environ.* 2023;31(2):793–803. 10.1080/10494820.2020.1808794

[ref13] Wang X Zhang L He T : Learning performance prediction-based personalized feedback in online learning via machine learning. *Sustainability.* 2022;14:7654. 10.3390/su14137654

[ref41] Williamson B Eynon R : Historical threads, missing links, and future directions in AI in education. *Learn. Media Technol.* 2020;45(3):223–235. 10.1080/17439884.2020.1798995

[ref43] Xia Q Chiu TKF Lee M : A Self-determination theory design approach for inclusive and diverse Artificial Intelligence (AI) K-12 education. *Comput. Educ.* 2022;189:104582. 10.1016/j.compedu.2022.104582

[ref6] Zawacki-Richter O Marín VI Bond M : Systematic review of research on artificial intelligence applications in higher education–where are the educators? *Int. J. Educ. Technol. High. Educ.* 2019;16:39. 10.1186/s41239-019-0171-0

[ref46] Zhai X : Practices and theories: How can machine learning assist in innovative assessment practices in science education. *J. Sci. Educ. Technol.* 2021;30(2):139–149. 10.1007/s10956-021-09901-8

