# Mapping the landscape: A bibliometric analysis of AI and teacher collaboration in educational research

**DOI:** 10.12688/f1000research.160297.2

**Published:** 2025-05-01

**Authors:** Arvind Nain, N.S Bohra, Archana Singh, Rekha Verma, Rakesh Kumar, Rajesh Kumar

**Affiliations:** 1 Department of management, Graphic Era Deemed to be University, Dehradun, Uttarakhand, India; 2 DBS Global University, Dehradun, Uttarakhand, India; 3 Uttaranchal Institute of Management, Uttaranchal University, Dehradun, Uttarakhand, India

**Keywords:** Artificial Intelligence, Teachers, Teaching, Education, Bibliometric

## Abstract

**Background:**

This study intends to investigate the relationship between artificial intelligence and teachers’ collaboration in educational research in response to the growing use of technologies and the current status of the field.

**Methods:**

A total of 62 publications were looked at through a systematic review that included data mining, analytics, and bibliometric methods.

**Result:**

The study shows a steady increase in the field of artificial intelligence and teacher collaboration in educational research, especially in the last few years with the involvement of the USA, China, and India. Education and information technology are the main contributors to this field of study, followed by an international review of open and distance learning research. The Scopus database was chosen for this study because of its extensive coverage of high-quality, peer-reviewed literature and robust indexing system, making it a dependable source for conducting bibliometric analyses. Scopus offers substantial information, citations tracking, and multidisciplinary coverage, which are critical for spotting publication trends, significant articles, major themes, and keywords in the area. The findings show that education and information technology make the most significant contributions to this sector, followed by international studies on open and distance learning.

**Conclusions:**

Over a three-year period, the average citation value is 12.44%. The education system, learning, e-learning, sustainability, COVID-19 issues, team challenges, organizational conflicts, and digital transformation are just a few of the topics it significantly contributes to. The study acknowledges its limitations and considers potential avenues for additional research. The results also emphasize important gaps in the literature, highlighting the necessity for more research. This information can help develop strategic approaches to address issues and take advantage of opportunities relating to artificial intelligence and teacher collaboration in higher education and research. The study’s ultimate goal is to offer guidance for tactics that promote teachers’ cooperation in educational research and the development of artificial intelligence.

## 1. Introduction

Artificial intelligence (AI) is a rapidly evolving technology with the potential to transform various aspects of society, including education. AI encompasses a wide range of capabilities, from natural language processing and machine learning to logical reasoning, sense-making, and problem-solving ( Smith & Humphreys, 2006; Chen et al., 2020). It has gained prominence in education through intelligent tutoring systems, chatbots, automated assessment tools, and personalized learning platforms that adapt to individual learning needs ( Wang et al., 2022; Metli, 2023). While AI offers numerous benefits in education, including personalized learning, administrative efficiency, and enhanced instructional methods, it also raises important concerns about the evolving role of educators. Teachers must adapt to technological advancements while preserving the fundamental principles of effective teaching, ethical considerations, and personal connections with students ( Pelletier et al., 2021; Zawacki-Richter et al., 2019).

The integration of AI in education often focuses on improving learning outcomes for students, but less attention has been given to how AI can enhance teacher collaboration within educational research. Teacher collaboration, which involves shared planning, co-authoring research, collaborative design, and knowledge dissemination, is a critical aspect of educational improvement. By facilitating seamless communication, data sharing, and co-creation of research materials, AI can potentially revolutionize how teachers collaborate on research projects.

Artificial Intelligence (AI) is a rapidly emerging technology that has the potential to alter many parts of society, including education. AI comprises of a wide range of features, including natural language processing, machine learning, logical reasoning, sense-making, and solving problems ( Smith & Humphreys, 2006; Chen et al., 2020). Automated tutoring systems, chatbots, computerized assessment tools, and customized educational platforms which adapt to student learning needs have all contributed to its popularity in education ( Wang et al., 2022; Metli, 2023). While AI has various benefits in education, such as tailored learning, increased administrative efficiency, and improved methods for teaching, it also raises serious questions about educators’ developing roles. Teachers must adapt to technology improvements while maintaining essential teaching concepts, ethical considerations, and personal ties among students ( Pelletier et al., 2021; Zawacki-Richter et al., 2019).

The incorporation of AI in education is frequently focused on enhancing student learning results, but less emphasis has been paid to how AI may improve teacher collaboration in educational research. Teacher collaboration, which includes coordinated planning, co-authoring research, collaborative design, and information sharing, is an important component of educational improvement. AI has the potential to alter the way teachers collaborate on research projects by allowing for seamless communication, data sharing, and collaborative creation of materials.

AI-enhanced teacher cooperation is especially important for linking research and practice because teachers are also consumers and providers of educational knowledge. However, research into how AI facilitates collaborative research efforts among teachers is still fragmented. The convergence of AI and teacher collaboration is sometimes dominated by technological developments and individual learning outcomes, rather than investigating the collaborative dynamics provided by AI tools.

Additionally, **Collaborative Learning Theory** suggests that knowledge is co-constructed through social interactions. AI tools can enhance these interactions by providing real-time feedback, personalized recommendations, and dynamic data analysis, thereby supporting collaborative research efforts.

However, the intersection between AI and teacher collaboration is often overshadowed by technical advancements and individual learning outcomes rather than examining the collaborative dynamics enabled by AI tools. This study aims to address this gap by exploring how AI can enhance teacher collaboration in educational research.

**Rationale for the Study**

Mapping the landscape of AI and teachers working together in educational research is vital given the growing use of AI in education and its significant effects on the processes of teaching and learning. The purpose of this bibliometric analysis is to shed light on the knowledge structure, research gaps, and current trends in this field. This study aims to identify trends and provide guidance for further research on AI and collaboration among teachers by reviewing the body of existing literature.

**Research Objectives**

1) To analyze the trends in publications over time about AI-enhanced teacher collaboration in the field of education.
2) To analyze affiliation production trends over time in the context of research on teacher collaboration and AI.
3) To use a word cloud and treemap analysis to illustrate important terms and ideas associated with AI-enhanced teacher collaboration.
4) To analyse worldwide partnerships and production patterns with AI and teacher collaboration studies.
5) To analyse the most recent and most popular subjects in educational research on AI-enhanced teacher collaboration.
6) To investigate the network of co-occurence of themes, authors, and keywords in research on teacher cooperation and AI.

## 2. Literature Review

### 2.1 AI-Enhanced Teacher Collaboration in Educational Research

AI applications in education have mostly focused on enhancing student learning results, optimizing administrative operations, and creating tailored experiences for students ( Chen et al., 2020; Zawacki-Richter et al., 2019). However, little emphasis has been placed on how AI may actively assist collaborative research and knowledge development among educators. Teachers frequently collaborate in educational research by co-authoring publications, designing research studies, exchanging data, and communicating findings. These collaborative behaviors are critical for professional growth, innovation, and knowledge construction ( Kusumastuti et al., 2023).

In this regard, AI offers exciting possibilities for enhancing teacher collaboration by providing advanced interaction platforms, automatic analysis of content, and intelligent systems for managing information. Tools powered by AI which include natural language processing (NLP) and algorithms based on machine learning are being increasingly incorporated through collaborative platforms to promote joint studies and simplifies knowledge sharing ( Luo et al., 2022).

Theoretical frameworks like Socio-Technical Systems (STS) and Community of Practice (CoP) are especially useful for understanding how AI might improve teacher cooperation. STS emphasizes the interaction of technological instruments (AI systems) and social systems (teacher cooperation), stressing how AI can function as a facilitator of collaboration dynamics rather than just a technical tool. From the CoP perspective, AI acts as a mediating tool, allowing multiple groups of educators to contribute knowledge, construct shared collections of research techniques, and maintain collaborative ties across time.

In addition, Collaborative Learning Theory suggests that AI-powered systems might improve real-time knowledge creation and co-authoring by offering continuous feedback, personal recommendations, and dynamic database analysis ( Boninger et al., 2020). However, while AI provides tools for improving cooperation, it does not substitute for educators’ ethical, social, and contextual judgments in research and practice.

### 2.2 Applications of AI in Teacher Collaboration

AI technologies are being used in a number of ways to improve teacher collaboration particularly in areas like:

1. **Co-Authoring and Collaborative Writing**: AI-assisted tools for writing and automated editing systems improve the efficiency and coherence of co-authored works ( Carvalho et al., 2022a, 2022b).
2. **Data Sharing and Analysis:** AI-based platforms facilitate collaboration of data analysis by offering real-time information, patterns identification, and predictive analytics to improve collective decision-making ( Luo et al., 2022).
3. **Knowledge Dissemination:** Automated content summarizing and proficient recommendation systems make it easier for educators to find and distribute important information.
4. **Collaborative Design of Research:** AI tools enhance brainstorming, mapping ideas, and planning projects easier, empowering teachers to co-design methods of research and instructional methodologies more effectively ( Kusumastuti et al., 2023).

Despite these advances, the available literature lacks a cohesive framework that explicitly links AI tools to collaborative research techniques among teachers. The research that do address teacher cooperation are primarily concerned with technical capabilities rather than social and organizational aspects. The dearth of complete based on theories research regarding how AI facilitates teacher cooperation poses a key void in the literature.

**Research Gap:**

The intersection between AI and teacher collaboration remains underexplored, with most studies prioritizing technical advancements over collaborative dynamics. Furthermore, current literature does not adequately address how AI tools influence the social processes of collaboration, particularly within educational research communities. By applying theories such as **Socio-Technical Systems, Communities of Practice, and Collaborative Learning Theory**, this study seeks to uncover the conceptual foundations and practical implications of AI-enhanced teacher collaboration.

Despite the expanding use of AI in research on education, the relationship of AI and teacher cooperation is still underexplored. Current research focuses mostly on technical elements, discrete educational effects, or unique AI applications. The joint dynamics of AI devices and human teachers, particularly in terms of knowledge and research development, have gotten little attention.

This work attempts to solve this gap by offering a full bibliometric assessment of AI-enhanced collaboration among teachers, finding publication trends, theme structures, and key contributions. While doing so, it intends to offer important insights into future study as well as practical use in educational contexts.

## 3. Methods

**Figure 1.**
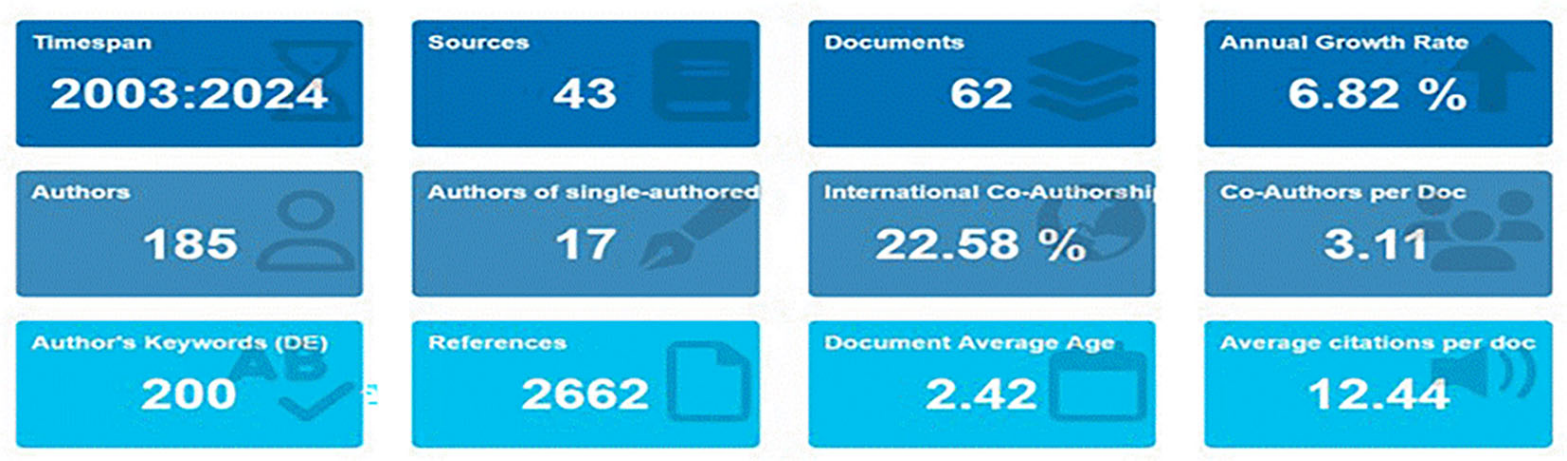
Main information. Source: BiblioShiny.

A bibliometric analysis aims to address specific research questions using a clear, systematic, and replicable search strategy ( Donthu et al., 2021). This process involves identifying relevant studies, synthesizing data, and analysing trends, such as the annual publication rates of articles ( Lin et al., 2013). The study focuses on exploring the literature on the use of artificial intelligence (AI) in education over the past 40 years, starting from 1984. The primary objectives are to answer the following questions: Which entities—such as research institutions, universities, countries, regions, and research communities—are the leading contributors to AI research in education? What is the intellectual; co Figure 1 displays the synthesised bibliometric data, which offers a comprehensive summary of the field’s current gaps or inconsistencies as well as research trends and real-world applications. This study provides insights into the issue and its evolution by methodically mapping 644 publications on AI in education fields and social framework of this research. Out of which a total of 62 publications were looked at through a systematic review that included data mining, analytics, and bibliometric methods.

### 3.1 Data Collection

In order to investigate how teacher cooperation and artificial intelligence (AI) connect in educational research, this study used bibliometric analysis. Scopus, a thorough and trustworthy academic database renowned for its wide coverage of peer-reviewed literature, provided the data. The research was conducted using words associated with “Artificial Intelligence,” “Teacher Collaboration,” “Educational Research,” along with other relevant terms. The inclusion criteria were confined to articles published in English during the previous two decades to ensure a complete assessment of the growing academic scene.

### 3.2 Data Analysis

The analysis involved two primary techniques: **Performance Analysis** and **Science Mapping**.

• Finding the most influential writers, sources, papers, and organizations that contribute to the field of study was the major goal of performance analysis.
• To depict the co-citation networks, thematic evolution, and intellectual structure of the literature, Science Mapping was carried out using programs like R bibliometrix and VOSviewer.

### 3.3 Ensuring Reliability of Coding and Analysis

For bibliometric assessment to produce reliable and repeatable results, coding and analysis dependability must be guaranteed. The following actions have been implemented to improve reliability:

**Use of Automated Tools:**

Well-known programs like VOSviewer and R bibliometrix, which are known for their precision in producing co-authorship, co-citation, and keyword co-occurrence networks, were used in the bibliometric analysis. By processing citation data using automated algorithms, these technologies reduce human bias.

**Data Cleaning and Preprocessing:**

The data extraction method required thorough filtering to exclude multiple entries, unnecessary articles, and sources which did not fulfil the inclusion criteria. To prevent bias, two researchers worked independently on the cleaning process.

**Inter-Coder Reliability Testing:**

To improve the reliability of theme code and classification, inter-coder accuracy was investigated. Two separate researchers carried out the coding technique and then examined their findings using Cohen’s Kappa coefficient. A total score of 0.75 or greater was deemed satisfactory, suggesting significant agreement among coders.

**Cross-Validation of Findings:**

The results generated from automated tools were cross-validated through manual checks to ensure consistency. Discrepancies were resolved through discussions and consensus between the researchers.

**Triangulation:**

The use of various tools (VOSviewer and R bibliometrix) provided the triangulation technique, which strengthened the findings by ensuring that the findings were consistent across different analytic approaches.

By implementing these reliability measures, the study aimed to ensure that the findings were both accurate and reproducible, thereby enhancing the credibility of the bibliometric review.

## 4. Results

### 4.1 Data Synthesis

Starting in 2003, this study looks at the literature on teacher cooperation and artificial intelligence (AI) in education during the previous 20 years. The following important questions are the focus of the study: Which organisations are the main contributors to research on artificial intelligence and teacher cooperation in education, including research institutes, universities, nations, regions, and research communities? Which conceptual, intellectual, and social frameworks are influencing this study? What changes have you seen over the years in the research of AI and teacher collaboration in education? A detailed summary of research trends and advancements in AI and teacher collaboration in education is shown in Figure 1, which presents the results of bibliometric analysis.

### 4.2 The Patterns of Article Publication Over Time

**Figure 2.**
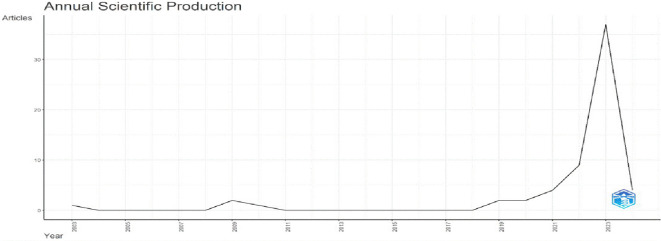
Annual scientific production. Source: BiblioShiny.

Figure 2 depicts the distribution of articles on AI and teacher collaboration in education published during the last 20 years (2003-2024). Research activity peaked in 2021 with 15 publications, but rose dramatically to 37 articles in 2023. Notably, the 2024 data only covers the first five months of the year, so the overall number of publications is projected to increase by the conclusion of the year. Even before 2022, the field had inconsistent research effort and changing publication outputs. However, the increased number of publications after 2021 indicates an increasing interest in the junction of AI and teacher cooperation. This growing trend implies that educators are becoming more aware of AI’s potential to improve collaborative research, knowledge dissemination, as well as and teaching practices. In addition, the average yearly growth rate for publication is 6.82 per year, indicating a gradual increase in research interest. The increasing citation numbers, however erratic, indicate that the field is increasingly gaining respect among the academic world. However, the data lacks a thorough examination of the research methodology used and the theoretical structures that underpin these investigations. It is critical to investigate if the increase in publications is accompanied by the development of strong theoretical models, particularly those relating to educational learning communities (TLCs) and professional learning networks (PLNs).

### 4.3 Source Growth

**Figure 3.**
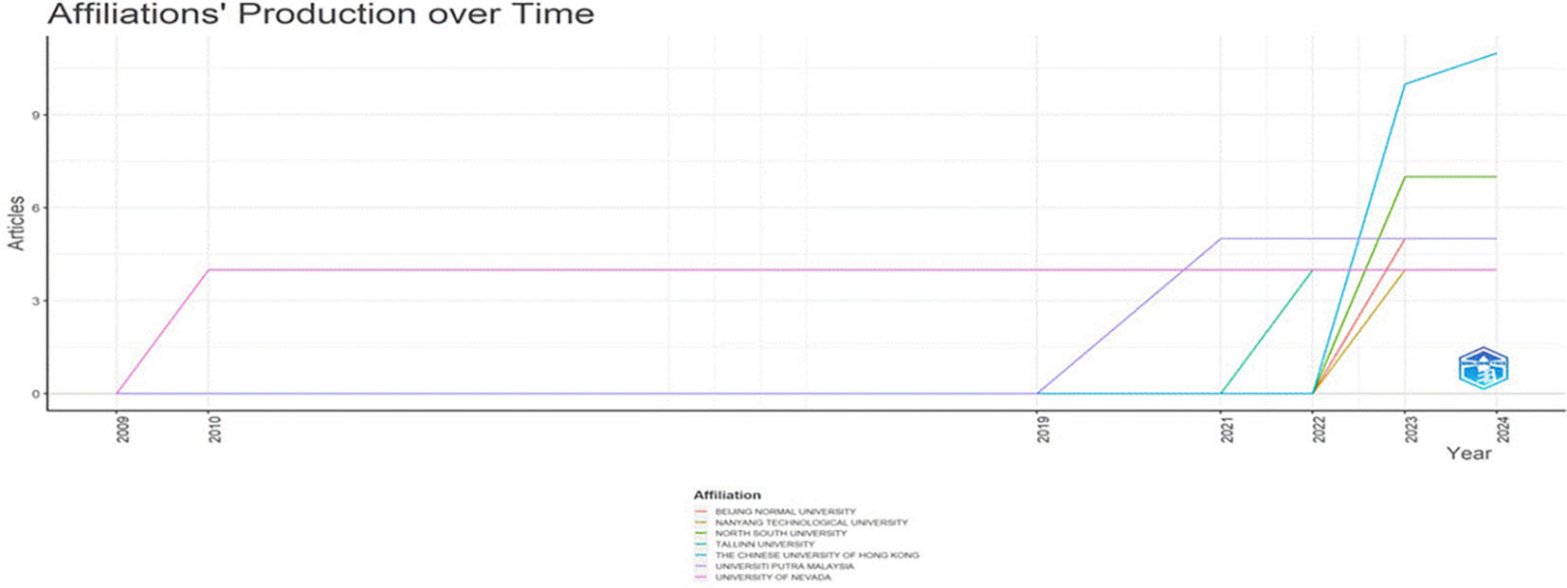
Affiliations production over time. Source: BiblioShiny.

The number of publications linked to different universities annually in Figure 3 shows journal contributions to research on AI and teacher cooperation in education. In the line chart, each university is represented by a different colour, and the study focusses on the top five because of their noteworthy contributions.

With publications in this area starting in 2022 and rising yearly after 2023, the Chinese University of Hong Kong and North South University have shown steady and significant development in their outputs. Since 2003, however, the other universities have contributed very little to this field of study.

### 4.4 Word Cloud, and Treemap

The rise in publications, particularly the prominences of terms like **“Artificial Intelligence,” “Teacher,” “Learning,” and “Students,”** indicates a growing interest in how AI can enhance educational processes. However, the focus appears heavily centered on technical applications and individual learning outcomes, with less emphasis on **collaborative practices** among educators.

**Figure 4.**
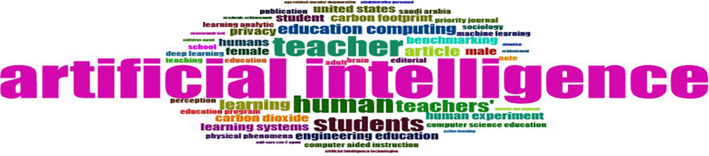
Word Cloud. Source: BiblioShiny.

For practice, this trend suggests that while AI tools are increasingly integrated into education, their application is primarily geared toward instructional efficiency and personalization rather than fostering collaborative research and knowledge sharing. For policy, the emphasis on **benchmarking, privacy, and computing** highlights concerns about standardization, ethical considerations, and technological accessibility.

**Research Methodologies:**

The word cloud reveals limited emphasis on theoretical frameworks or collaborative methodologies. The frequent appearance of **“Publication” and “Article”** suggests a descriptive, publication-focused approach, likely involving **bibliometric analysis, systematic reviews, or conceptual studies**. However, the lack of terms directly related to qualitative or empirical research suggests that **methodological diversity is lacking**.

**Theoretical Frameworks:**

The word cloud does not prominently feature established theoretical frameworks like **Teacher Learning Communities (TLCs)** or **Professional Learning Networks (PLNs)**. This absence implies that most studies have not grounded their analysis in collaboration theories, which could have provided deeper insights into how AI supports or hinders collaborative dynamics among educators.

### 4.5 Word Growth

**Figure 5.**
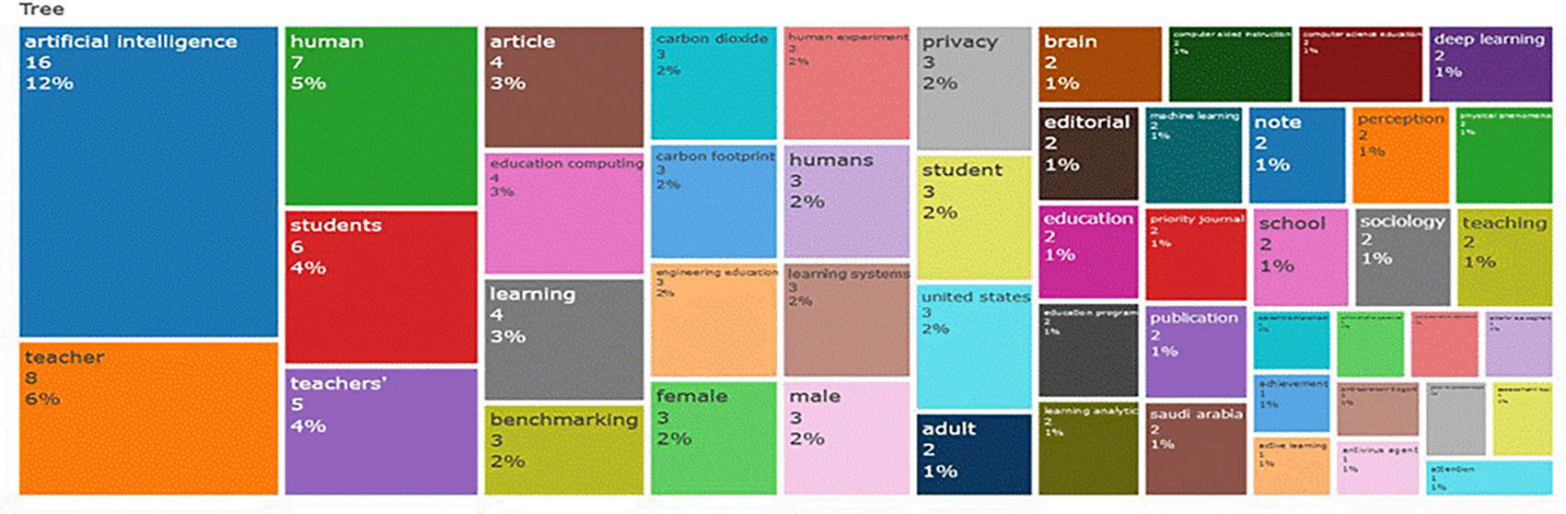
Treemap. Source: BiblioShiny.

Examining word growth provides important information on how literary terminology has changed throughout time. As shown in Figure 5 AI and teacher cooperation in education, where understanding the development of the field is aided by monitoring the introduction and impact of key phrases. learning system, employee, students, and educational computing are some of the first terms in this domain. For professionals, analysts, and researchers, this analysis is an essential tool that helps them spot patterns and gain insightful knowledge. Analysing word frequency over time reveals trends that can help influence strategic planning and decision-making in a variety of situations.

### 4.6 Country-specific Production and Collaborations

**Figure 6.**
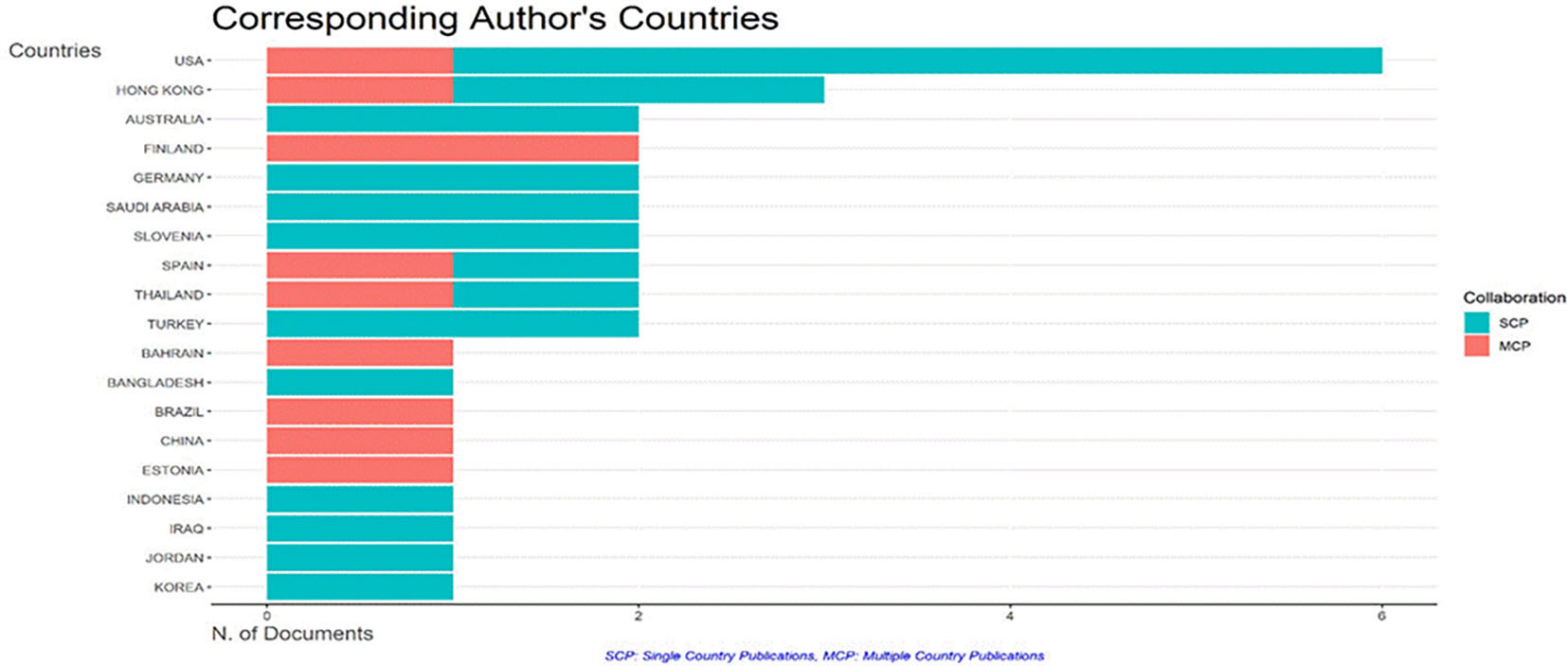
Most productive country. Source: BiblioShiny.

The cooperation globe map ( Figure 6) emphasizes international collaborations related to AI and teacher collaboration research in education. The United States and Hong Kong emerge as the most influential states, with the strongest international linkages. This partnership pattern is most likely impacted by their superior research infrastructure, funding availability, and the active participation of academics from prestigious universities such as the Chinese University in Hong Kong and North South University.

The Growing contributions from these nations indicate a growing organizational interest in AI-enhanced research in education. Still, the dominance of certain nations raise concerns about the worldwide scope of study in this sector. Future research should investigate if research results from underrepresented nations are absorbed into the larger discourse, or whether there is a geographical bias in AI-related teacher cooperation research.

**Interpretation of Trends & Implications:**

While descriptive patterns provide useful insights into the total number and distribution of publications, further study is required to identify the implications for policy, practice, and research approaches.

**Practical Implications:**

The rapid increase in papers reflects rising recognition of AI’s potential to improve teacher collaboration. However, do these collaborations improve educational methods or only serve academic interests?

How well are AI tools used in collaborative studies to enhance instructional tactics and learning outcomes?

**Policy Implications:**

The global importance of the United States and Hong Kong means that AI-related educational initiatives may be heavily influenced by research from these locations.

Policies that focus on encouraging international collaboration may improve the variety and inclusion of research results.

**Nature of Studies Analyzed:**

The studies examined by BiblioShiny are mostly descriptive and analytical, with an emphasis on charting patterns and trends rather than doing experimental research. The methodology utilized in this bibliometric analysis is helpful in recognizing research gaps and significant contributions, however it may fall short in terms of investigating why AI is important for teacher cooperation.

### 4.7 Trend Topics

**Figure 7.**
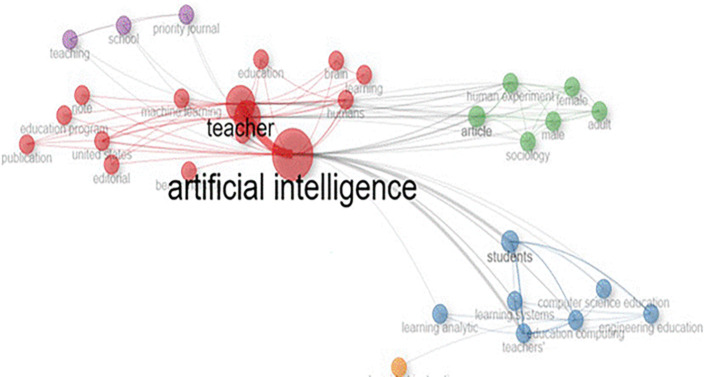
Co-occurrence network. Source: Vosviwer.

A quantitative analysis of the influence of scholarly literature is shown in Figure 7, possibly using bibliometric techniques. This assessment takes into account measures like publications and citations, offering a quantifiable indication of the impact and prominence of research results. Important terms like “Artificial Intelligence,” “Teacher,” “Teachers,” “Students,” and “human,” which probably reflect major issues in the literature, are included in the study. According to the results, “Artificial Intelligence” became the most popular topic, especially in 2023, indicating a major emphasis on AI and its cooperation with educators.

This demonstrates how research trends are dynamic and underscores the necessity of continuous observation. It is advised that researchers remain alert to new developments and modify their focus to meet changing goals and problems.

The quantitative analysis presented in Figure 7 **(Co-occurrence network)** and Table 1 **(Word frequency per year)** highlights the growing emphasis on **Artificial Intelligence (AI)** in educational research, particularly in 2023. The prominence of terms like **“Teacher,” “Teachers’,” “Students,” and “Human”** suggests that the integration of AI is being discussed in relation to **educational stakeholders (teachers and students)** and how AI can potentially transform learning and teaching practices.

**Table 1.** Word frequency per year.

item	freq	year_q1	year_med	year_q3
teacher	8	2018	2022	2023
students	6	2022	2022	2023
artificial intelligence	16	2022	2023	2023
human	7	2022	2023	2023
teachers’	5	2023	2023	2023

However, these findings remain **descriptive rather than analytical**. While it’s clear that AI is a hot topic, the **practical and policy-related implications** are not addressed. For instance:

• What specific **AI tools or systems** are being applied to enhance teacher collaboration?
• How does the **interaction between AI and teachers** influence teaching practices, curriculum design, or research activities?
• Are AI tools promoting collaborative learning frameworks like **Teacher Learning Communities (TLCs)** or **Professional Learning Networks (PLNs)?**

**Implications for Practice:**

The rise in publications suggests a **growing interest in exploring AI’s role in education**, but mostly from a technological rather than a collaborative standpoint. For **educators and practitioners**, understanding how AI tools facilitate **co-authoring, knowledge sharing, and collaborative research design** could significantly enhance collaborative practices.

**Implications for Policy:**

Policymakers need to **address gaps** in the integration of AI with **teacher collaboration practices**. Clear guidelines and frameworks are needed to promote ethical, effective, and equitable use of AI in educational research collaborations.

**Research Methodologies:**

The word cloud reveals limited emphasis on theoretical frameworks or collaborative methodologies. The frequent appearance of **“Publication” and “Article”** suggests a descriptive, publication-focused approach, likely involving **bibliometric analysis, systematic reviews, or conceptual studies**. However, the lack of terms directly related to qualitative or empirical research suggests that **methodological diversity is lacking**.

**Theoretical Frameworks:**

The word cloud does not prominently feature established theoretical frameworks like **Teacher Learning Communities (TLCs)** or **Professional Learning Networks (PLNs)**. This absence implies that most studies have not grounded their analysis in collaboration theories, which could have provided deeper insights into how AI supports or hinders collaborative dynamics among educators.

### 4.8 The Co-occurrence Network Analysis

**Different Interpretation of Co-occurrence Network Analysis:**

The co-occurrence network analysis reveals **different thematic streams within AI-teacher collaboration research**, represented by color-coded clusters. This clustering helps identify **key themes and their relationships** based on the frequency and proximity of keywords. However, the interpretation lacks **content-based synthesis and thematic analysis**, which are essential for deeper insights.

***Central Themes & Connections (Red Cluster):***

• The **red cluster**, considered central due to its high centrality, indicates **core topics** most frequently discussed within the literature.
• Terms like **“Artificial Intelligence,” “Teacher,” and “Collaboration”** likely appear here, suggesting that studies predominantly focus on **how AI is integrated into teacher collaboration practices.**

***Thematic Relationships (Purple & Green Clusters):***

• The **interconnecting purple and green clusters** indicate concepts that frequently appear together, suggesting **conceptual overlap**.
• These clusters likely involve themes such as **AI-driven tools for professional learning, co-authoring platforms, and collaborative research design.**
• Potentially linked to **Collaborative Learning Theory and Communities of Practice (CoP)**, where AI tools facilitate the **development of shared practices and knowledge exchange**.

***Isolated Themes (Yellow-Orange & Grey Clusters):***

• These clusters are more discrete, indicating **niche topics or emerging areas of research**.
• They may represent **less integrated or less frequently studied topics**, such as **AI for professional development networks or specialized AI applications for instructional design**.
• The weak connections suggest **limited exploration of how these themes are integrated into mainstream AI-teacher collaboration studies**.

**Research Gaps & Missing Analysis:**

The analysis does not thoroughly examine:

1. **Thematic Gaps:** Why are certain clusters isolated? Are there missed opportunities to connect AI applications across various educational practices?
2. **Types of Collaborations Supported by AI:** The analysis should specify **what kinds of collaborations AI is enhancing:**
  ○ **Research Partnerships:** Co-authoring tools, collaborative research frameworks.
  ○ **Co-Teaching:** AI-driven platforms enabling joint lesson planning and instructional design.
  ○ **Professional Development Networks:** AI tools supporting **Teacher Learning Communities (TLCs)** or **Professional Learning Networks (PLNs)**.
3. **Theoretical Integration:** Application of theories like **Socio-Technical Systems (STS)** and **CoP** could explain how **AI tools enable or hinder various types of teacher collaboration**.

**Suggestions for Improvement:**

• Conduct a **content-based synthesis** of the most frequently occurring keywords in each cluster.
• Identify how AI tools are used in **specific types of collaboration** and whether these applications are **theoretically grounded**.
• Explore potential **conceptual overlaps between clusters** and suggest areas for **future interdisciplinary research**.

## 5. Discussion

This study indicates that while this industry’s publication count has grown consistently since 2019, a bigger growth occurred in 2024, which was followed by an exponential rise after 2019. In terms of AI and teacher collaboration in Education, the United States far outpaces China. The recent publication of numerous highly referenced articles suggests an increase in the quantity of academic publications on the function of AI and teacher collaboration in Education as well as a change in the focus of some of this research. As a consequence, the industry seems to be developing significantly and moving forward. Researchers are now working to have a better understanding of how AI and teacher collaboration could influence Education more sustainably. The results of this study show that authorship and institutional location affect the level of interest in AI and teacher collaboration in Education on a worldwide scale. Unexpectedly, the US has the most authors and colleges, with Hongkong coming in second. The data clearly shows the geographic variety of the AI and teacher collaboration in Education in terms of institutional affiliations and authorships. Geographic variety is important because different areas may have different views on the role that AI should play with teachers in education. The fact that the research suggests that the focus is still on how AI functions in teacher collaboration in respond to various educations programmes inside educational institute or whether particular AI system have an influence on narrowly defined education system is a potentially worrisome aspect. Less focus has been paid to distance educations and the importance of sustainability in AI and teacher collaboration in Education systems, which emphasises how challenging it is to incorporate sustainability into Education.

### Practical Implications

The results of this bibliometric analysis are useful for various kinds of stakeholders in the fields’ education.

Educators: Understanding AI tools’ collaborative potential can help improve teaching efficacy by reducing administrative duties, personalizing learning experiences, and delivering real-time feedback. Educators may use AI technologies to build more dynamic and welcoming educational environments, which improves overall teaching quality.

Policymakers: The study’s insights may aid in the development of policies that govern the ethical use of AI within educational environments, providing access to AI educational resources while dealing with adoption barriers. Interdisciplinary cooperation between AI specialists and teachers can be facilitated by creating guidelines which policymakers can put into place.

AI Developers: Understanding collaborative teaching challenges, AI developers can create new technologies that include adaptive learning systems, automated evaluation systems, and AI-based tools to help teachers maximize student participation and achievement.

These practical implications underline the need for a synergistic approach including educators, policymakers, and AI developers to create productive AI-teacher collaboration and enhance educational methods.

## 6. Conclusion

Through a systematic review, this study delved into the realm of artificial intelligence and teacher collaboration in Education. The research revealed a growing interest in the field and a diverse range of applications of artificial intelligence technologies, highlighting the need for a comprehensive examination of their utilization from various perspectives. The findings underscored the significant reliance on artificial intelligence technologies, pointing towards a future shaped by algorithmic scenarios. Drawing upon the insights garnered from the reviewed publications, the study identified several implications for future research endeavors. Firstly, it was observed that a majority of the artificial intelligence applications in Education predominantly focus on technical aspects, disregarding crucial factors such as pedagogy, curriculum, and instructional/learning design. Secondly, despite the utilization of human-generated data in artificial intelligence technologies, there is a notable absence of regulations regarding the ethical usage of this data. To address this gap, future research could concentrate on exploring this issue and advocating for the development of policies and strategies. Educational institutions must prioritize the establishment of a human-centered approach to online learning that effectively harnesses the benefits of artificial intelligence technologies.

### Ethics and consent

Ethical approval and consent were not required.

## Data Availability

Zenodo: Mapping the Landscape: A Bibliometric Analysis of AI and Teacher Collaboration in Educational Research. Doi: 10.5281/zenodo.14706394 ( https://doi.org/10.5281/zenodo.14706394) ( Nain, 2025). This project contains the following extended data:
• Mapping the Landscape A Bibliometric Analysis of AI and Teacher Collaboration in Educational Research.csv Data is available under the terms of the Creative Commons Zero v1.0 Universal

## References

[ref1] Adiguzel T Kaya MH Cansu FK : Revolutionizing education with AI: Exploring the transformative potential of ChatGPT. *Contemp. Educ. Technol.* 2023;15(3). 10.30935/cedtech/13152

[ref2] Alves JL Nadae J de Carvalho MM de : Knowledge management enablers and barriers: exploring the moderating effect of communication barriers. *Int. J. Manag. Proj. Bus.* 2022;15(7):1091–1122. 10.1108/IJMPB-02-2022-0047

[ref3] Boninger F Molnar A Salda˜na C : *Big claims, little evidence, lots of money: The reality behind the Summit Learning Program and the push to adopt digital personalized learning platforms.* Boulder, CO: National Education Policy Center;2020. nepc.colorado.edu/publication/summit-2020.

[ref4] Bozkurt A Karadeniz A Baneres D : Artificial intelligence and reflections from educational landscape: A review of AI studies in half a century. *Sustainability (Switzerland).* 2021;13(2):1–16. 10.3390/su13020800

[ref5] Carvalho L Martinez-Maldonado R Tsai YS : How can we design for learning in an AI world? *Comput. Educ.: Artif. Intell.* 2022a;3(February):100053. 10.1016/j.caeai.2022.100053

[ref6] Carvalho RP Marchiori CFN Brandell D : Artificial intelligence driven in-silico discovery of novel organic lithium-ion battery cathodes. *Energy Storage Mater.* 2022b;44(October 2021):313–325. 10.1016/j.ensm.2021.10.029

[ref7] Chen X Xie H Zou D : Application and theory gaps during the rise of artificial intelligence in Education. *Comput. Educ.: Artif. Intell.* 2020;1:100002. 10.1016/j.caeai.2020.100002

[ref8] Chiu TKF : A holistic approach to Artificial Intelligence (AI) curriculum for K- 12 schools. *TechTrends.* 2021;65:796–807. 10.1007/s11528-021-00637-1

[ref9] Chiu TKF Meng H Chai CS : Creation and evaluation of a pre-tertiary Artificial Intelligence (AI) curriculum. *IEEE Trans. Educ.* 2022;65(1):30–39. 10.1109/TE.2021.3085878

[ref10] Chiu TKF Xia Q Zhou X : Systematic literature review on opportunities, challenges, and future research recommendations of artificial intelligence in education. *Comput. Educ.: Artif. Intell.* 2023;4(December 2022):100118. 10.1016/j.caeai.2022.100118

[ref11] Donthu N Kumar S Mukherjee D : How to conduct a bibliometric analysis: An overview and guidelines. *J. Bus. Res.* 2021;133:285–296. 10.1016/j.jbusres.2021.04.070

[ref12] Dutt A Ismail MA Herawan T : A systematic review on educational data mining. *IEEE Access.* 2017;5:15991–16005. (accessed 22 July 2019). 10.1109/ACCESS.2017.2654247 Reference Source

[ref13] Feng X Wei Y Pan X : Academic emotion classification and recognition method for large-scale online learning environment—Based on A-CNN and LSTM-ATT deep learning pipeline method. *Int. J. Environ. Res. Public Health.* 1941;17:17. 10.3390/ijerph17061941 PMC7142864 32188094

[ref14] Gardner H : Frames of mind: The theory of multiple intelligences. *Basic Books.* 1985.

[ref15] Holmes W Tuomi I : State of the art and practice in AI in education. *Eur. J. Educ.* 2022;57(4):542–570. 10.1111/ejed.12533

[ref16] Hussain S : Education 4.0 made simple: Ideas for teaching. *Int. J. Literacy Educ.* 2018;6(3):92–98. 10.7575/aiac.ijels.v.6n.3p.92

[ref17] Jiménez-Hernández D González-Calatayud V Torres-Soto A : Digital competence of future secondary school teachers: Differences according to gender, age, and branch of knowledge. *Sustainability (Switzerland).* 2020;12(22):1–16. 10.3390/su12229473

[ref18] Karaca O Çalışkan SA Demir K : Medical artificial intelligence readiness scale for medical students (MAIRS-MS) – development, validity and reliability study. *BMC Med. Educ.* 2021;21(1):112–119. 10.1186/s12909-021-02546-6 33602196 PMC7890640

[ref19] King J Holmes R Burkholder S : Advancing nature-based solutions by leveraging Engineering With Nature ^®^ strategies and landscape architectural practices in highly collaborative settings. *Integr. Environ. Assess. Manag.* 2022;18(1):108–114. 10.1002/ieam.4473 34101357

[ref20] Kusumastuti R Silalahi M Sambodo MT : Understanding rural context in the social innovation knowledge structure and its sector implementations. *Manag. Rev. Q.* 2023;73(4):1873–1901. 10.1007/s11301-022-00288-3

[ref21] Lin CF Yeh Y Hung YH : Data mining for providing a personalized learning path in creativity: An application of decision trees. *Comput. Educ.* 2013;68:199–210. 10.1016/j.compedu.2013.05.009

[ref22] Luo Y Han X Zhang C : Prediction of learning outcomes with a machine learning algorithm based on online learning behaviour data in blended courses. *Asia Pac. Educ. Rev.* 2022;25:267–285. 10.1007/s12564-022-09749-6

[ref23] Maaten L van der Hinton G : Visualizing data using t-SNE. *J. Mach. Learn. Res.* 2008;9:2579–2605.

[ref24] Metli A : Articles on Education and Artificial Intelligence: A Bibliometric Analysis Articles on Education and Artificial Intelligence: A Bibliometric Analysis. *J. Soc. Sci. Educ. (JOSSE).* 2023;6:279–312. 10.53047/josse.1352197

[ref25] Mhlanga D : The Role of Artificial Intelligence and Machine Learning Amid the COVID 19 Pandemic: What Lessons Are We Learning on 4IR and the Sustainable Development Goals. *Int. J. Environ. Res. Public Health.* 2022;19(3). 10.3390/ijerph19031879 35162901 PMC8835201

[ref26] Muñoz JLR Ojeda FM Jurado DLA : Systematic Review of Adaptive Learning Technology for Learning in Higher Education. *Eurasian J. Educ. Res.* 2022;2022(98):221–233. 10.14689/ejer.2022.98.014

[ref27] Nain A : Mapping the Landscape: A Bibliometric Analysis of AI and Teacher Collaboration in Educational Research.[Data set]. *Zenodo.* 2025. 10.5281/zenodo.14706394 PMC12082066 40386461

[ref28] Nemorin S Vlachidis A Ayerakwa HM : AI hyped? A horizon scan of discourse on artificial intelligence in education (AIED) and development. *Learn. Media Technol.* 2023;48(1):38–51. 10.1080/17439884.2022.2095568

[ref29] Nigam A Pasricha R Singh T : A systematic review on ai-based proctoring systems: Past, present and future. *Educ. Inf. Technol.* 2021;26(5):6421–6445. 10.1007/s10639-021-10597-x 34177348 PMC8220875

[ref30] Papadopoulos I Koulouglioti C Papadopoulos C : *Transcultural Artificial Intelligence and Robotics in Health and Social Care.* Academic Press;2022.

[ref31] Pelletier K Brown M Brooks DC : Educause Horizon Report Teaching and Learning Edition. *Educause.* 2021. (accessed on 18 December 2022). Reference Source

[ref32] Prinsloo P : Fleeing from Frankenstein’s monster and meeting Kafka on the way: Algorithmic decision-making in higher education. *E-Learn.* 2017;14:138–163.

[ref33] Rai A Mishra A : The Role of Artificial Intelligence in the Automation of Human Resources. *Adoption and Implementation of AI in Customer Relationship Management.* Jan 2022. 10.4018/978-1-7998-7959-6.ch011

[ref34] Smith AE Humphreys MS : Evaluation of unsupervised semantic mapping of natural language with Leximancer concept mapping. *Behav. Res. Methods.* 2006;38:262–279. 10.3758/BF03192778 16956103

[ref35] Stadlman M Salili SM Borgaonkar AD : Artificial Intelligence Based Model for Prediction of Students’ Performance: A Case Study of Synchronous Online Courses During the COVID-19 Pandemic. *J. STEM Educ.* 2022;23(2):39–46. Reference Source

[ref36] Wang C : Emotion recognition of college students’ online learning engagement based on deep learning. *Int. J. Emerg. Technol. Learn.* 2022;17:110–122. 10.3991/ijet.v17i06.30019

[ref37] Wang S Christensen C Cui W : When adaptive learning is effective learning: comparison of an adaptive learning system to teacher-led instruction. *Interact. Learn. Environ.* 2023;31(2):793–803. 10.1080/10494820.2020.1808794

[ref38] Wang X Zhang L He T : Learning performance prediction-based personalized feedback in online learning via machine learning. *Sustainability.* 2022;14:7654. 10.3390/su14137654

[ref39] Williamson B Eynon R : Historical threads, missing links, and future directions in AI in education. *Learn. Media Technol.* 2020;45(3):223–235. 10.1080/17439884.2020.1798995

[ref40] Xia Q Chiu TKF Lee M : A Self-determination theory design approach for inclusive and diverse Artificial Intelligence (AI) K-12 education. *Comput. Educ.* 2022;189:104582. 10.1016/j.compedu.2022.104582

[ref41] Zawacki-Richter O Marín VI Bond M : Systematic review of research on artificial intelligence applications in higher education–where are the educators? *Int. J. Educ. Technol. High. Educ.* 2019;16:39. 10.1186/s41239-019-0171-0

[ref42] Zhai X : Practices and theories: How can machine learning assist in innovative assessment practices in science education. *J. Sci. Educ. Technol.* 2021;30(2):139–149. 10.1007/s10956-021-09901-8

