# Supplementary material for: Mapping the landscape: A bibliometric analysis of AI and teacher collaboration in educational research
Source: F1000Res. 2025 May 1;14:201. Originally published 2025 Feb 13. [Version 2] doi: 10.12688/f1000research.160297.2 (PMC12082066; doi:10.12688/f1000research.160297.2)
Supplement: Peer review history

# Reviewer report: Reviewer response for version 2

**DOI:** 10.5256/f1000research.180509.r381969

**Published:** 2025-05-12

**Authors:** Musa Adekunle Ayanwale

**Affiliations:** 1 University of Johannesburg, Auckland Park, Gauteng, South Africa

The authors have addressed my previous concerns. Thank you.

Is the work clearly and accurately presented and does it cite the current literature?

Yes

If applicable, is the statistical analysis and its interpretation appropriate?

Partly

Are all the source data underlying the results available to ensure full reproducibility?

Yes

Is the study design appropriate and is the work technically sound?

Partly

Are the conclusions drawn adequately supported by the results?

Yes

Are sufficient details of methods and analysis provided to allow replication by others?

Partly

Reviewer Expertise:

AI in Education, Psychometrics, Educational Measurement, Test development, and Computer Adaptive Testing

I confirm that I have read this submission and believe that I have an appropriate level of expertise to confirm that it is of an acceptable scientific standard.

# Reviewer report: Reviewer response for version 1

**DOI:** 10.5256/f1000research.176170.r366717

**Published:** 2025-03-03

**Authors:** Musa Adekunle Ayanwale

**Affiliations:** 1 University of Johannesburg, Auckland Park, Gauteng, South Africa

The authors address an emerging and significant area of educational research: the intersection of AI and teacher collaboration. While the topic is timely, the manuscript lacks a focused, methodologically transparent, and critically reflective analysis.

 1. The research objectives should be more precise; for example, are the authors more interested in how AI enhances teacher collaboration in research or how AI supports collaborative teaching? These are distinct questions.

 2. The introduction and literature review should narrow the focus to highlight why teacher collaboration is particularly important in the context of AI.

 3. The literature review is overly broad, covering AI in education generally, without sufficiently focusing on the collaborative aspect between teachers in educational research or practice.

 4. The conceptual connection between AI and teacher collaboration is not well-established. Currently, the link is implied but not explicitly justified with clear theoretical underpinnings.

 5. Citations are mostly descriptive (what AI can do) rather than analytical (why AI matters for teacher collaboration). A deeper engagement with collaboration theories (e.g., teacher learning communities, professional learning networks) would significantly improve this section.

 6. The search and inclusion criteria are underdeveloped. The authors claim to have reviewed 62 papers, but the database search strategy is not transparent enough. Key details missing include the exact search strings used, the rationale for choosing Scopus only, and whether grey literature or conference proceedings were excluded; if so, why?

 7. There was no discussion on how the reliability of coding or analysis was ensured, which is critical in bibliometric reviews.

 8. While the descriptive trends (publication counts, country collaborations, keyword frequencies) are well presented by the authors, they lack meaningful interpretation. What does the rise in publications actually mean for practice or policy? What are the dominant research methodologies being used? What theoretical frameworks underpin these papers?

 9. Co-occurrence analysis is underexplored; the clusters are described superficially without discussing the thematic gaps between clusters. Additionally, there is no content-based synthesis of the papers. What kinds of collaborations (research partnerships, co-teaching, professional development networks) are being supported by AI? This would add value.

Is the work clearly and accurately presented and does it cite the current literature?

Yes

If applicable, is the statistical analysis and its interpretation appropriate?

Partly

Are all the source data underlying the results available to ensure full reproducibility?

Yes

Is the study design appropriate and is the work technically sound?

Partly

Are the conclusions drawn adequately supported by the results?

Yes

Are sufficient details of methods and analysis provided to allow replication by others?

Partly

Reviewer Expertise:

AI in Education, Psychometrics, Educational Measurement, Test development, and Computer Adaptive Testing

I confirm that I have read this submission and believe that I have an appropriate level of expertise to confirm that it is of an acceptable scientific standard, however I have significant reservations, as outlined above.

# Reviewer report: Reviewer response for version 1

**DOI:** 10.5256/f1000research.176170.r366716

**Published:** 2025-03-03

**Authors:** Amar Johri

**Affiliations:** 1 Saudi Electronic University, Riyadh, Riyadh Province, Saudi Arabia

Dear Editor,

 Thank you very much for assigning the manuscript "Mapping the landscape: A bibliometric analysis of AI and teacher collaboration in educational research" for review.

 The study addresses an important and emerging area—the intersection of AI and teacher collaboration in education. I appreciate the author's hard work in conceptualizing and designing this study. I have carefully examined the study and must appreciate the commendable work done by the authors. I have the following minor observations:

 1. The introduction section is nicely written. The authors should briefly explain the rationale behind the study in this section. The introduction should clearly state the study’s main research questions or objectives. 

 2. The literature review is well crafted. A summary should highlight the major gap between the previous published literature and the present study. 

 3. The research methodology and results are clearly presented. Using bibliometric analysis to map research trends is a solid methodological choice.

 4. The authors used only one database, Scopus. A brief explanation should be provided on why the Scopus database is preferred.

 4. A brief highlight on practical implications should explain how the findings could benefit educators, policymakers, and AI developers.

 I endorse this manuscript for indexing—best wishes to the authors.

Is the work clearly and accurately presented and does it cite the current literature?

Yes

If applicable, is the statistical analysis and its interpretation appropriate?

Yes

Are all the source data underlying the results available to ensure full reproducibility?

Yes

Is the study design appropriate and is the work technically sound?

Yes

Are the conclusions drawn adequately supported by the results?

Yes

Are sufficient details of methods and analysis provided to allow replication by others?

Yes

Reviewer Expertise:

General Management, Bibliometric analysis

I confirm that I have read this submission and believe that I have an appropriate level of expertise to confirm that it is of an acceptable scientific standard.
